# Relaxing the restricted structural dynamics in the human hepatitis B virus RNA encapsidation signal enables replication initiation in vitro

**DOI:** 10.1371/journal.ppat.1010362

**Published:** 2022-03-08

**Authors:** Katharina Dörnbrack, Jürgen Beck, Michael Nassal

**Affiliations:** Department of Internal Medicine II/Molecular Biology, University Hospital Freiburg, Freiburg, Germany; The Pennsylvania State University College of Medicine, UNITED STATES

## Abstract

Hepadnaviruses, including hepatitis B virus (HBV) as a major human pathogen, replicate their tiny 3 kb DNA genomes by capsid-internal protein-primed reverse transcription of a pregenomic (pg) RNA. Initiation requires productive binding of the viral polymerase, P protein, to a 5´ proximal bipartite stem-loop, the RNA encapsidation signal ε. Then a residue in the central ε bulge directs the covalent linkage of a complementary dNMP to a Tyr sidechain in P protein´s Terminal Protein (TP) domain. After elongation by two or three nucleotides (nt) the TP-linked DNA oligo is transferred to a 3´ proximal acceptor, enabling full-length minus-strand DNA synthesis. No direct structural data are available on hepadnaviral initiation complexes but their cell-free reconstitution with P protein and ε RNA (Dε) from duck HBV (DHBV) provided crucial mechanistic insights, including on a major conformational rearrangement in the apical Dε part. Analogous cell-free systems for human HBV led at most to P—ε binding but no detectable priming. Here we demonstrate that local relaxation of the highly basepaired ε upper stem, by mutation or via synthetic split RNAs, enables ε-dependent in vitro priming with full-length P protein from eukaryotic translation extract yet also, and without additional macromolecules, with truncated HBV miniP proteins expressed in bacteria. Using selective 2-hydroxyl acylation analyzed by primer extension (SHAPE) we confirm that upper stem destabilization correlates with in vitro priming competence and show that the supposed bulge-closing basepairs are largely unpaired even in wild-type ε. We define the two 3´ proximal nt of this extended bulge as main initiation sites and provide evidence for a Dε-like opening of the apical ε part upon P protein binding. Beyond new HBV-specific basic aspects our novel in vitro priming systems should facilitate the development of high-throughput screens for priming inhibitors targeting this highly virus-specific process.

## Author summary

Chronic hepatitis B virus (HBV) infection puts >250 million people at an increased risk for severe liver disease. Current treatments can control but rarely cure infection. HBV features a 3,200 bp DNA genome, generated by reverse transcription of a pregenomic (pg) RNA. To initiate DNA synthesis the viral polymerase, P protein, employs a stem-loop on pgRNA, ε, to covalently link a defined first nucleotide to its Terminal Protein (TP) domain. This protein-priming is highly virus-specific yet poorly understood. More is known for duck HBV (DHBV) where, different from HBV, protein-priming was successfully reconstituted in vitro years ago. One insight was that gaining priming-competence involves opening of the apical stem in DHBV ε RNA (Dε); in HBV ε the more extensive basepairing might restrict such dynamics. Here we relaxed these constraints by identifying functional but less stably folded, including split, HBV ε variants. Several such variants supported in vitro priming, including in a simple two-component-system employing a shortened recombinant P protein. Amongst other data the new cell-free systems yielded a first view on a major conformational change in HBV ε RNA bound to P protein, highlighting the importance of RNA dynamics for the human virus. Beyond furthering basic understanding our data should facilitate screening for protein-priming inhibitors as new anti-HBV agents.

## Introduction

**Fig 1:**
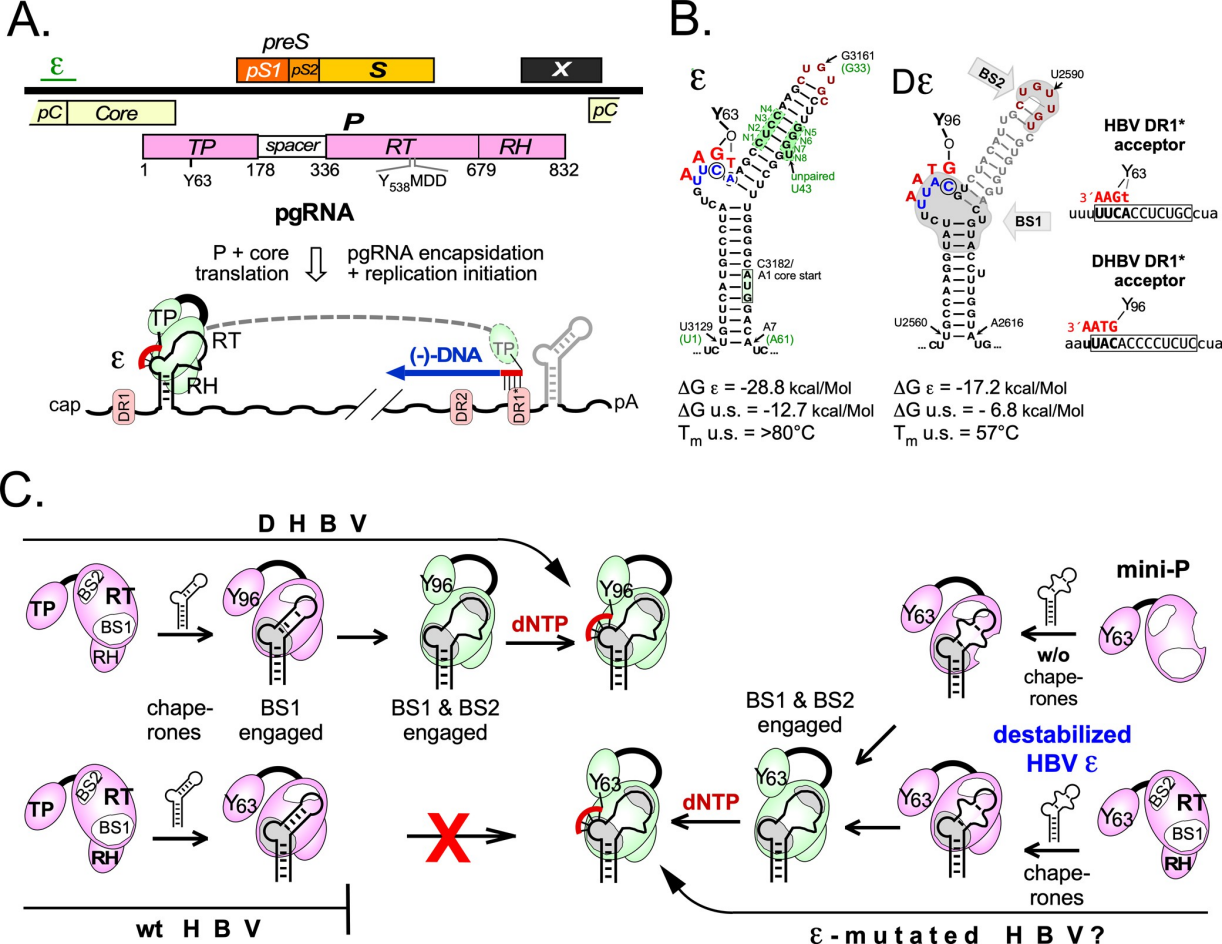
Basic features of HBV replication. **(A) Linearized version of the HBV genome.** The 3.2 kb genome is symbolized by the black line, ORFs by colored bars; ε shows the location of the RNA encapsidation signal. S, surface antigen; X, HBx; P, polymerase; pC, preC; pS1, preS1, pS2, preS2. Domains of P protein with approximate borders (in amino acid positions) are denoted as TP (Terminal Protein), RT (Reverse Transcriptase) and RH (RNase H), Y63 refers to the Tyr acceptor for the first primer nucleotide, Y538MDD to RT active site residues. Terminally redundant pg RNA mediates translation of P and core. P binding to 5´ ε initiates pgRNA encapsidation and synthesis of a DNA oligo (red) that after transfer to an acceptor site at direct repeat DR1* is extended into full-length minus-strand DNA (blue); plus-strand DNA synthesis and degradation of the copied RNA yield partially double-stranded RC-DNA. **(B) Secondary structures of HBV ε and DHBV ε (Dε).** The structures and free energies (ΔG) for the entire stem-loops and just the upper stems (u.s.) are as predicted by M-FOLD, supplemented by experimental melting temperatures (T_m_) for just the upper stems [38]. For ε black numbers refer to genome positions relative to the starting A of the core ORF as position 1; green numbers to positions within the stem-loop only. Y63 and Y96 denote the priming HBV and DHBV TP Tyr residues. Dε regions BS1 and BS2 (grey background) are likely interaction sites with DHBV P [30]. The DR1* acceptor sites for the (D)ε templated primers are shown on the right. **(C) Hepadnaviral protein priming.** The model is based on in-cell and in vitro data for DHBV. Accordingly, chaperones activate P for Dε binding via interaction with BS1 (bulge region). Only a subsequent interaction with BS2 (UG-rich apical sequence) and conformational rearrangements in the RNA enable P (symbolized by the green coloring) to initiate DNA synthesis at Y96. The central hypothesis of this study is that the P-ε complex from HBV, unlike that from DHBV, is unable to progress, in vitro, beyond BS1 binding because the higher stability of the upper stem impairs the necessary rearrangement. Mutations that reduce basepairing but maintain ε functionality might enable DHBV analogous in vitro priming systems, including chaperone-independent formats based on truncated miniP proteins.

Chronic infection with hepatitis B virus (HBV) affects >250 million people worldwide, and the associated liver diseases cause up to 900,000 deaths per year [1]. Approved therapeutics, i.e. type-I interferon (IFN) and select nucleos(t)ide analogs (NAs), may control but rarely cure infection [2,3]. HBV, the prototypic hepadnavirus (*hepa**totropic* *DNA* virus), is a small enveloped virus that replicates its tiny 3.2 kb DNA genome through reverse transcription of a pregenomic (pg) RNA (Fig 1A), initiated by protein-priming rather than conventional nucleic acid priming [4]; full-length DNA synthesis occurs only inside nucleocapsids [5]. These peculiar features would lend themselves as highly specific therapeutic targets; however, in the absence of feasible, high-throughput compatible assays mechanistic knowledge of protein-priming by human HBV is limited. Most concepts outlined below are based on data from the duck HBV (DHBV) model virus, especially from cell-free reconstitution of DHBV protein-priming, first realized by in vitro translation of DHBV polymerase (P protein) in rabbit reticulocyte lysate (RRL) [6]; as yet, attempts to set up an analogous completely cell-free system for the human virus have failed.

The infectious cycle of all hepadnaviruses starts with envelope protein-mediated entry of virions into the target cell (for review: [7]), release of the nucleocapsid (core particle) into the cytoplasm, and delivery of the capsid-borne partially double-stranded (ds) relaxed circular (RC-) DNA into the nucleus; there it is converted, likely by host DNA repair factors [8], into covalently closed circular (ccc) DNA [9,10], the template for new viral transcripts. These comprise subgenomic mRNAs for the envelope proteins and, in mammalian HBVs, for HBx, an epigenetic regulator of cccDNA transcriptional activity [11]; and the greater-than-genome length pgRNA (Fig 1A) plus the precore RNA from which the secretory hepatitis B e antigen (HBeAg) is derived [12]. PgRNA acts as mRNA for the capsid (core) protein and P protein, and in addition as precursor for a new DNA genome. RNA to DNA conversion requires binding of P protein to the encapsidation signal ε, a 5´-proximal ~60 nt stem-loop structure [13,14] on pgRNA (Fig 1A and 1B). Formation of the P- ε ribonucleoprotein (RNP) complex recruits core protein dimers and thus triggers pgRNA encapsidation [12], and it initiates protein-primed reverse transcription. There a tyrosyl hydroxyl group in P´s unique Terminal Protein (TP) domain (Y63 in HBV, Y96 in DHBV; Fig 1B) becomes covalently attached to a first deoxynucleotide, templated by a 3´ proximal nt in the bulge of the (D)ε stem-loop and catalyzed by P protein´s reverse transcriptase (RT) domain. Limited extension on the ε bulge yields a 3–4 nt oligonucleotide which, still bound to TP, is translocated to an acceptor at the 3´ direct repeat, DR1* (Fig 1A and 1B). Extension from there and concomitant degradation of pgRNA by P´s RNase H (RH) domain generate single-stranded (ss) minus-strand DNA. The 5´ terminal pgRNA residues are spared from degradation and prime plus-strand DNA synthesis. Besides some double-stranded linear (dsL) DNA the main product is the hepadnavirus-typical RC-DNA with P protein on the minus-strand 5´ end and RNA on the plus strand 5´ end [4,5]. This protein-primed reverse transcription is an ancient mechanism of genome replication [15].

While the general pathway from pgRNA to RC-DNA can be monitored for HBV and DHBV in virus replicating cells, in vitro reconstituted DHBV priming provided unique mechanistic insights, including the role of 5´ Dε as replication origin for minus-strand DNA [16–18], as genetically confirmed for HBV [19]. Replacing in vitro translation by addition to RRL of extraneously generated P protein [20,21] identified other priming-relevant components, in particular chaperones [22–25], and defined Dε binding and priming-relevant residues within P protein [26,27]. Conversely, short Dε stem-loop transcripts, including non-natural nucleic acid derivatives [28], enabled definition of the respective determinants in the RNA [29,30]. Truncated yet initiation-competent DHBV P protein derivatives (miniP) led to simpler, chaperone-independent priming assay formats [23,31], requiring just miniP protein, Dε RNA, dNTPs and proper buffer conditions. In sum this led to a mechanistic model (Fig 1C, *top left*) whereby chaperones activate P for ε binding, and the RNA triggers the enzymatic activity of P, similar to the RNAs in Cas nucleases [32]. In vitro priming assays even provided proof-of-principle that small compounds can inhibit the DHBV priming reaction [33,34]. HBV P protein, however, has never shown strictly ε-dependent priming activity in any such system, and neither when recombinantly expressed in insect cells [35] or multi-step purified from *E*. *coli* [36].

Various factors could be responsible for these failures. Generating recombinant P protein in soluble, active form is already difficult for DHBV and could be even more problematic for the HBV enzyme. Second, HBV P protein may have more elaborate co-factor requirements than DHBV P [23]; however, in vitro translating HBV P in extracts from human HeLa or HepG2 hepatoma cells rather than RRL did not, in our hands, lead to detectable priming activity. A third and under-investigated factor is the ε RNA itself, more precisely its structure and capability for structural dynamics. As free RNAs, the known hepadnavirus ε elements can take on a similar bipartite structure (Fig 1B) with a lower and an upper stem, a central bulge (containing the priming template) and an apical loop [13,14,37,38]. Gross alterations, e.g. deletion of the bulge or loop, generally disrupted both ε and Dε function in virus-replicating cells, and impaired DHBV in vitro priming activity. This has long shaped the notion that a stable stem-loop structure is critical for ε function yet several observations with DHBV challenge this view. Structure probing of free vs. P protein-bound Dε RNA revealed an opening of the upper stem which strictly correlated with priming activity [39]. Systematic evolution of ligands by exponential enrichment (SELEX) for DHBV P binding RNAs yielded several Dε variants with reduced base-pairing in the upper stem that remained priming-proficient in vitro [40] and supported infection in vivo [41]; even marked variations in length and sequence of the upper Dε stem did not block in vitro priming and replication as long as a few sequence-determinants (including a G-C or G-U pair following the bulge plus an apical GUUGU motif) were present in accessible form [30].

From this we proposed a two-step priming activation model (Fig 1C, *top left*) [30,39] whereby initial P binding involves the bulge and its closing base-pairs, termed binding site 1 (BS1); a subsequent rearrangement in the apical stem then enables the GUUGU motif to engage with P protein at BS2. This model is supported by P-binding yet priming-inactive Dε variants with a preserved bulge but mutated GUUGU motif, e.g. variant S2 [40,41]. The model also fits the ability of recombinant HBV P to specifically bind its cognate ε in vitro, yet without gaining priming activity [42]; such non-productive binding was independent of apical ε subelements, e.g. the loop, which are indispensable for the P-ε interaction in virus replication [42]. Also none of various HBV ε variants from a SELEX for in vitro HBV P protein binding displayed in vitro priming activity [43]. Hence these variants apparently contained a bulge-encompassing interaction site for initial P protein binding but lacked the apical site(s) required to form a productive RNP complex. Similarly, these sites, while physically present, might be unavailable in wild-type HBV ε RNA if the extensive basepairing in the upper stem prevents progression into a new, more open priming-competent structure (Fig 1C, *bottom*). In Dε the energy for reshaping the upper stem is much lower [38,40,44], hence the rearrangement may be less dependent on accessory factors than with HBV. A role for such factors is suggested by the currently most advanced cell-free HBV priming system wherein FLAG-tagged HBV P protein and ε RNA are co-expressed in transfected HEK293T cells [45,46]. The in-cell formed RNP complexes are enriched by immunoprecipitation, then supplemented with appropriate buffer and α^32^P-dNTPs. While successfully used to define interaction determinants in P protein and ε [47,48] the complexity of the system makes it unsuitable for high-throughput applications. Most importantly, cellular co-expression of the ε RNA is mandatory for RNP formation, as neither post-isolation RNA loading nor exchange of intracellularly loaded RNA on P protein seem possible. Hence each combination of P protein and/or ε RNA must individually be co-expressed and purified [45,49], suggesting that cellular factors are required for productive RNP formation.

We here aimed to develop a simpler, more versatile and completely cell-free HBV priming system by enhancing structural flexibility in the upper ε stem (Fig 1C, right). As various mutations in this region impeded the interaction with P protein in cells [14,42,49] we adapted an in-cell selection procedure [30] to screen for replication-competent HBV ε sequences. These should comply with all steps of pgRNA reverse transcription; hence P binding-competent but priming-defective variants [40,42,43] would be excluded. From a library of HBV expression plasmids comprising 8 randomized positions in the ε upper stem (N1 to N8 in Fig 1B) we characterized some 20 replication-competent non-wt ε sequences. Importantly, some mutants supported ε-dependent in vitro priming with full-length HBV P protein translated in RRL yet also with bacterially expressed HBV miniP protein in a chaperone-independent system. We show that synthetic ε RNAs carrying destabilizing nicks or gaps in the upper stem are suitable templates for HBV in vitro priming, and we provide first evidence for a major conformational rearrangement of the ε upper stem in P protein-bound vs. free HBV ε RNA.

Together our data confirm that the highly stable secondary structure of HBV ε is key to previous failures in reconstituting HBV protein-priming in vitro, and they provide access to feasible two-component assays for assessing the impact of modifications of P protein, ε RNA as well as exogenous factors on this activity. This should pave the way for the identification of inhibitors targeting this highly specific step in the HBV life-cycle.

## Results

### In-cell selection of replication-competent yet structurally destabilized HBV ε variants

Key to validating our hypothesis was access to less stably folded but functional HBV ε RNA variants. As the appropriate extent of destabilization was unknown and previous studies had not revealed a systematic map of mutation-tolerant sites within ε [13,14,42,50,51] we employed a SELEX procedure to explore many ε variants in parallel for their ability to support HBV DNA formation in cells when part of a complete pgRNA. Such RNA sequences must be capable of a productive interaction with P protein. Adapting an in-cell-SELEX method established for DHBV [30], outlined in S1 Fig, we generated pools of HBV expression vectors in which pgRNA transcription is driven by the cytomegalovirus immediate-early enhancer/promoter ("CMV promoter") as in the parental wild-type (wt) HBV vector pCH-9/3091 [52], however, with randomized positions N1 to N8 in the ε upper stem (nominally 65.536 variants; S1A Fig). This region comprises three of the six stabilizing G-C pairs (Fig 1B), and its counterpart in Dε is rather tolerant towards mutations [30]. Upon transfection into human hepatoma cells only pgRNAs carrying functional ε elements would be packaged and reverse transcribed (S1B Fig). PCR amplification of viral DNAs from intracellular nucleocapsids and/or secreted virions should then exclusively yield functional ε sequences, especially upon reiteration of the selection procedure. Conversely, too many cycles might restrict selection to few sequences with a replication advantage, such as perhaps wt ε.

To minimize the risk of contamination with wt ε during HBV vector pool cloning the viral genomes in the recipient plasmids were genetically tagged (e.g. pCH-9_190), or had the 5´ ε region plus ~500 bp downstream replaced by a large stuffer fragment and a deletion in 3´ ε (S1A Fig). The resulting starting vector pool was indeed highly degenerate at the 8 target positions (pCH-9/3093_Δ3´ε_PCR2; S2 Fig). When transfected into Huh7 cells two independent aliquots (a, b) both produced intracellular capsids which contained HBV DNA, as shown by native agarose gel electrophoresis (NAGE) and subsequent blotting (S2A Fig). Signals were approximately one tenth as intense as those from wt HBV vector pCH-9/3091, suggesting the pool contained at least a fraction of pgRNAs with functional ε elements. This was corroborated by Southern blot analysis of the DNAs from intracellular capsids and extracellular particles (S2A Fig), which in all cases revealed 3.2 kb dsL DNA as major product. Near-genome-length PCR (comprising 5´ ε and ~2.8 kb downstream sequence) generated wt-like patterns of full-length plus two prominent shorter products, identified as derived from the known pgRNA splicing products SP1 and SP3 [53]; this also excluded transfected plasmid DNA as a major template in the PCRs. Direct sequencing of the pool-derived amplicons showed heterogeneous nt compositions at seven of the randomized positions, yet virtually only T at N8 (S2B Fig), the authentic unpaired U43 in wt ε RNA. Recloning the amplicon ε regions yielded a new vector pool for transfection, and the whole procedure was repeated three more times. After the second round, in addition to N8 (T) positions N2 (T) and N7 (G) displayed a clear enrichment of the wt nt. After the third round, the wt nt predominated at all positions except N5; and after the fourth round only wt sequence was detectable (S2B Fig). Hence DNA from the first three rounds appeared as most promising source for functional non-wt ε sequences. We isolated from 15 to 24 individual clones from each of rounds 1 to 3, and an additional 12 clones from round 4 plus 10 clones from the unselected pool. A first sequence inspection revealed, consistent with the pool sequencing, an increasing proportion of wt sequence, from 1 in 10 for the starting pool over 2 in 15, 8 in 24 and 9 in 22 after rounds 1 to 3, to 12 in 12 after round 4. Most of the totally 51 non-wt clones carried 4 to 7 mutations.

**Fig 2:**
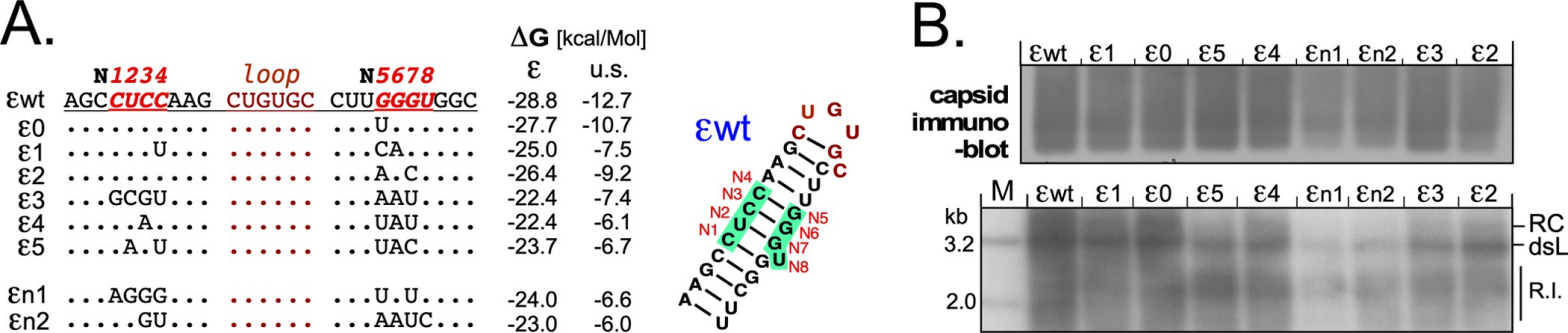
In-cell SELEX derived HBVs with non-wild-type ε are replication competent. **(A) Sequences of individual ε variants tested.** Randomized positions N1 to N8 (red letters) are depicted in the context of the wt ε upper stem, non-wild-type nt are indicated for each variant. The last columns show the calculated ΔG values (M-FOLD) for the most stable predicted full-length (ε) and upper stem (u.s.) structures; for reference, the secondary structure of the wt ε upper stem is also shown. Variant ε1 combined few mutations (3), good replication competence and reduced ΔG, and was chosen for further experiments. **(B) Formation of capsids and replicative DNA intermediates by select variants.** Individual HBV plasmids carrying the indicated ε sequences were transfected into Huh7 cells alongside wt HBV vector pCH-9/3091. Four days post transfection, cytoplasmic extracts were analyzed for capsids by NAGE and subsequent immunoblotting (*top*), and for viral DNA from cytoplasmic nucleocapsids by Southern blotting, using a ^32^P-HBV DNA probe (*bottom*); R.I., replicative intermediates. The overall patterns were for all variants similar to those from wt HBV, in line with the bulk HBV DNA content of the intracellular capsids, and the appearance of replicative intermediates in extracellular viral particles shown in S3 Fig.

M-Fold predicted secondary structures could be broadly categorized into wt-like with wt-like stabilities (ΔG = -28.8 to -26.0 kcal/Mol), and those with lower stability (ΔG = -25.0 to -21.2 kcal/Mol), often with two or more energetically similar predicted structures. In a first pilot validation of replication-competence by transfection of 40 individual miniprep DNAs about one third did not produce DNA containing capsids, in several cases owing to mutations outside the randomized positions, likely from the multiple PCR steps. We hence focussed on provenly functional clones covering a variety of ε sequences with differing numbers of mutations and predicted structures and free energies (ε0 to ε5, εn1, εn2; Fig 2); for instance, the predicted ΔG for the single-site mutant ε0 was -27.7 kcal/Mol and thus similar to wt ε (-28.8 kcal/Mol), that for the seven-site variant ε3 was only -22.9 kcal/Mol. As shown by native agarose gel electrophoresis (NAGE) followed by anti-HBc immunoblotting all clones generated intracellular capsids, although for εn1 and εn2 in somewhat reduced amounts (Fig 2, right, *top panel*). Southern blotting of the isolated capsid DNAs revealed for all clones a similar pattern of full-length and less mature replicative intermediates, again with the lowest intensities for samples εn1 and εn2 (Fig 2, right, *bottom panel*). This semiquantitative ranking was confirmed by direct detection of HBV DNA in NAGE separated capsids and Southern blotting of DNA from extracellular particles (S3 Fig); signal intensities there suggest a positive correlation of wt-like DNA amounts with fewer mutations and higher stability (ε0, see above; and ε2 with two mutations and ΔG = -26.4 kcal/Mol), in line with the rapid selection of wt ε during SELEX. Importantly, however, non-wt ε sequences with up to seven mutations and substantially lowered upper stem stability could productively interact with HBV P protein in cells, despite the high conservation of the ε sequence amongst natural HBV isolates [54].

### Structure probing confirms local destabilization in the upper stem of select ε variants

**Fig 3:**
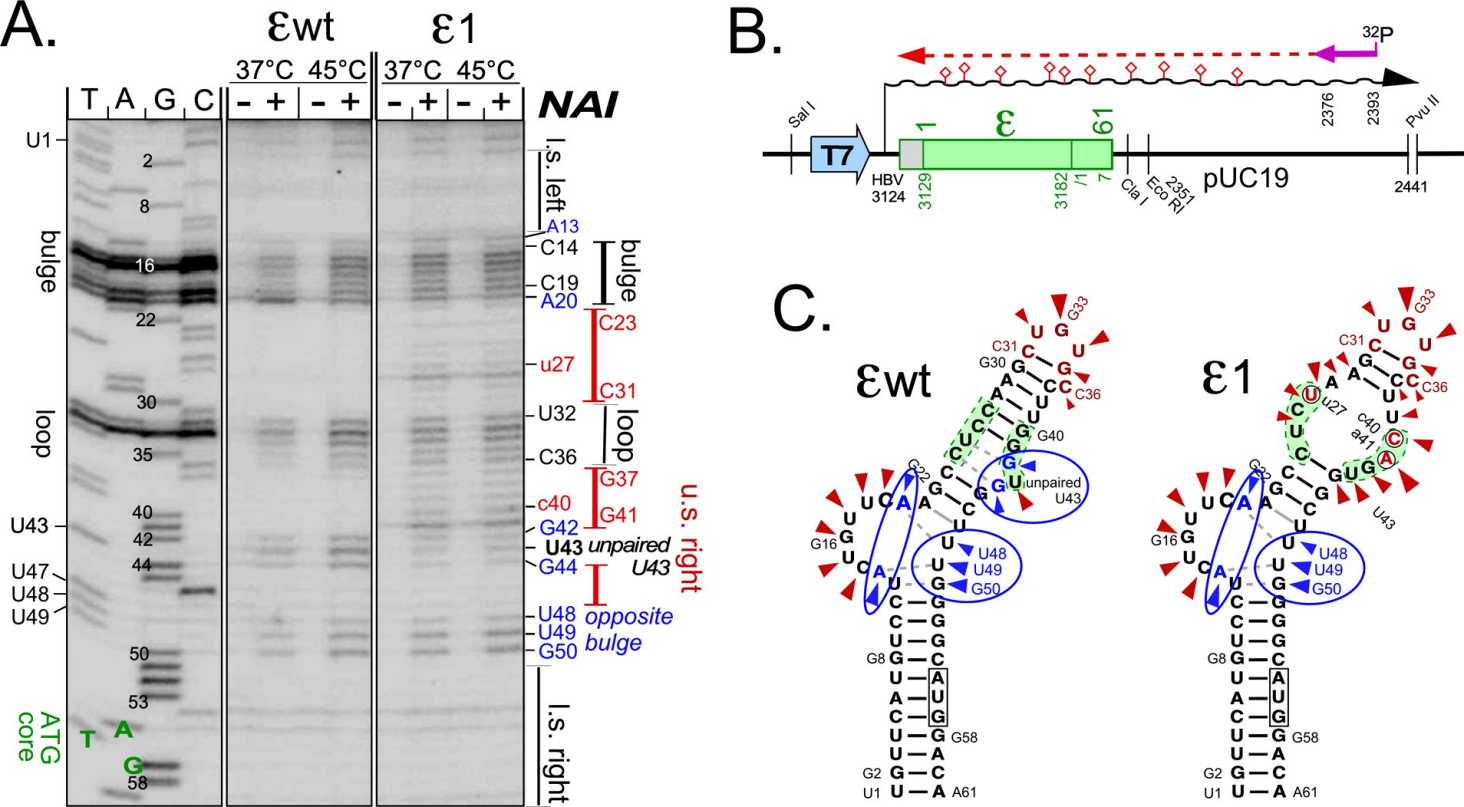
SHAPE reveals more open than expected bulge and unpaired U43 regions in wt ε RNA and confirms reduced upper stem stability by ε1 mutations. **(A) Analysis of SHAPE primer extension products by denaturing polyacrylamide gel electrophoresis (PAGE).** RNAs produced as outlined in (B) were reacted with 2-methylnicotinic acid imidazolide (NAI) at 37°C and 45°C, and modified sites were mapped by primer extension using a 5´-^32^P labeled primer; a dideoxy sequencing ladder on the pUC19T7 plasmid harboring the wt ε sequence served as marker. Borders of the classical ε subelements are indicated on the right. **(B) Assay scheme.** Pvu II linearized pUC19T7 plasmids [30] harboring the respective ε sequences were used as templates for T7 RNA polymerase run-off transcripts; NAI modified RNAs were then subjected to primer extension. NAI modified sites (diamonds) prevent progress of the extension polymerase. **(C) SHAPE-adjusted secondary structure models.** NAI hits at accessible 2´ hydroxyls are indicated by arrowheads. For wt ε RNA hits expected from the classical model are shown in red; additional hits in blue. Accordingly, the bulge itself (A13, A20), the region opposite the bulge (U48, U49, G50) and the G residues neighboring the unpaired U43 were more accessible than expected from enzymatic probing (see S4 Fig for a direct comparison plus SHAPE data for 20°C and 60°C). The additional hits in ε1 RNA fit the anticipated opening of the central upper stem, with the strongest hit near U43 shifted to the mutant A at the G41 position (indicated as a41).

For wt ε the second-most likely predicted structure is ~6 kcal/Mol less stable than that shown in Fig 1B, suggesting the latter structure is highly likely to prevail. In contrast, for various selected variants different structures within a narrow free energy window were predicted. For ε1 (ΔG = -25.0 kcal/Mol), for instance, the second- and third-most stable structures had ΔG values of -24.9 and -24.1 kcal/Mol. As ε1 also carried only three mutations it was chosen for experimental structure comparison with wt ε. Confirming earlier data [13] enzymatic probing using the single-strand (ss) specific RNases A (pyrimidine-specific) and T2 (base-non-specific) revealed strong bulge (U15) and loop (U32) signatures in both RNAs, indicating a common stem-bulge-stem-loop architecture (S4A Fig). Additional RNase hits around the mutated sites in ε1 (C27U, G40C) indicated a more open structure although the same region was also cleaved by V1 nuclease which recognizes double-helical RNA and stacked bases that are not canonically paired [55,56]. Hence these nt may adopt such a structured but nonpaired conformation, or they underly local structural dynamics between alternative conformations. To overcome the intrinsic limitations of enzymatic probing, we employed Selective 2´-Hydroxyl acylation Analyzed by Primer Extension (SHAPE; Fig 3); there, a small electrophilic molecule such as 2-methylnicotinic acid imidazolide (NAI) targets the RNA backbone in less constrained regions [57]. NAI-modified sites cause polymerase stops during a subsequent primer extension reaction (Fig 3B). The increased resolution of SHAPE versus enzymatic probing is illustrated in S4B Fig.

For wt ε as well as ε1 four similarly accessible regions were revealed, namely the bulge plus A20, the loop, the unpaired U43 and its neighboring G42 and G44 residues, and nt U48-U49-G50 opposite the bulge (Fig 3A and 3C). Notably, such a more open structure was not evident by enzymatic mapping, except that RNase T2 also cut at A13 (S4A Fig), classically drawn as part of the lower stem´s top base pair.

The absence of specific signals between residues G1—U12 and G51—C60 in both ε wt and ε1 further indicated the presence of a stable lower stem in both RNAs. However, ε1 RNA yielded extra bands upstream (U25 –A29) and downstream the loop (C37 –A41), in line with the desired local destabilization in the center of the upper stem. SHAPE analyses at different temperatures (shown in Fig 3A for 37°C and 45°C, and in S4B Fig additionally for 20°C and 60°C) affected band intensities (stronger at higher temperature) but not the patterns as such. These data confirmed ε1 as a prototypic non-wt ε RNA combining functionality in HBV replication with the desired local destabilization of the upper stem structure.

### Select upper stem-destabilized ε variants support HBV in vitro priming in RRL

**Fig 4:**
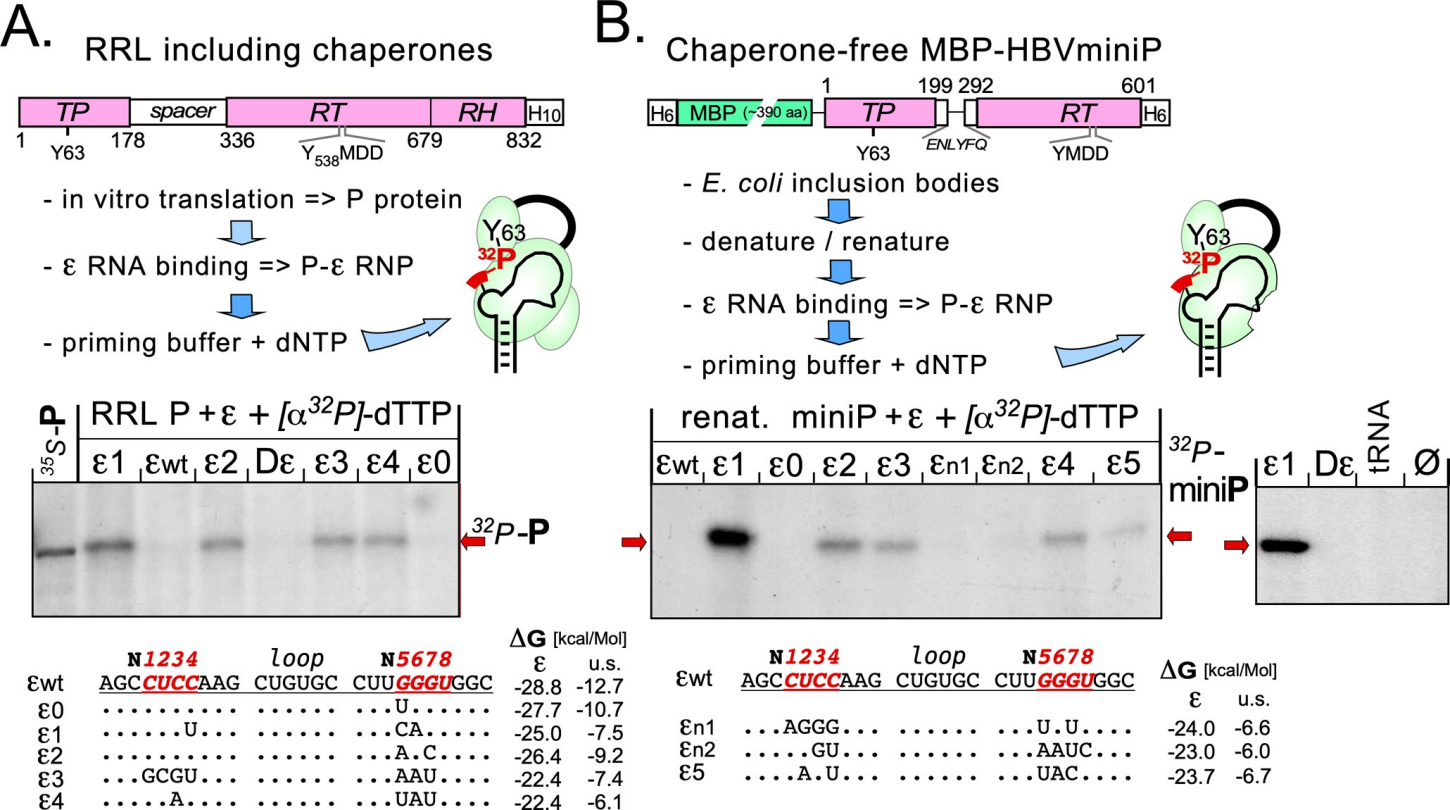
Select ε upper stem mutations enable in vitro priming. **(A) RRL system.** RRL programmed with full-length HBV P RNA in vitro translates P protein and provides chaperones. Next ε RNA is added for RNP formation. Finally priming conditions are established by adding priming buffer which includes bivalent metal ions such as Mg^2+^ and/or Mn^2+^ plus the desired α^32^P-dNTP (here dTTP). Successful priming results in ^32^P labeled P protein, detected by SDS-PAGE followed by autoradiography. ^35^S-labeled P protein produced by in vitro translation served as size marker. The most stably folded RNAs, ε wt and ε0, as well as Dε gave virtually no signal while ε1 to ε4 did. **(B) Chaperone-free HBV miniP system.** The bacterial HBV miniP expression construct ending after residue I601 was designed by homology to a priming-active DHBV miniP construct [26]. The fusion protein was massively expressed as inclusion bodies and refolded as described for DHBV miniP and shown in S5 Fig. RNP formation and priming were induced as in RRL. Neither Dε nor tRNA nor a reaction without added RNA gave a comparable signal to ε1 RNA (*right panel*).

With ε1 plus several additional RNAs with *bona fide* similar properties (ε2 - ε5) at hand we next tested their ability to support in vitro priming in comparison with wt ε and the replication-selected stably folded single-site mutant ε0. We started with full-length HBV P protein translated in RRL, owing to the extract´s established role in promoting specific, though non-productive, binding of HBV ε to HBV P protein [42]. Priming activity of in vitro transcribed wt and variant ε RNAs was assessed by addition of [α^32^P]dTTP, with the A following the bulge (A20 in Fig 3) as one of the assumed template nt [19,58]; a detailed analysis of initiation site selection is presented below. Successful priming results in ^32^P-labeling of P protein (Fig 4A). Confirming previous failures no ^32^P-labeled P protein was detectable with wt-ε RNA, and neither with ε0 nor Dε; however, variants ε1 to ε4 all produced bands with virtually identical mobility as the ^35^S-Met labeled HBV P protein used as marker (Fig 4A). The inactivity of Dε supports specificity of the reaction, and that of ε0 suggests that loss of one basepair in the upper stem is insufficient to enable in vitro priming (as corroborated below). Together these data demonstrated the validity of our SELEX strategy and they support the hypothesis that the high stability of the upper stem in wt ε prevents conversion into a new, productive conformation.

### A cell factor-independent HBV in vitro priming system

We next aimed for a simpler system that would maintain the crucial features of authentic protein-priming. We and others have previously shown that appropriately truncated DHBV P proteins, e.g. DHBV miniP with a deletion of the downstream part of the RT and the RH domain after position 575, exert Dε dependent but chaperone independent priming activity; hence miniP, Dε RNA, dNTPs and suitable buffer conditions are sufficient for a functional priming assay [23,26,27,31].

We therefore designed an analogous HBV miniP protein (Fig 4B) in which ~100 residues in the nonessential spacer region are substituted by a short peptide, and the C terminal residues of the RT domain downstream position 601 plus the RH domain are replaced by a His-tag (Fig 4B); the C terminal border was derived from a multiple alignment of hepadna- and nackednavirus P proteins [59] whereby the homologous residue to W575 in DHBV P is I601 in HBV P. To facilitate soluble expression in E. coli, we added a maltose-binding-protein (MBP) tag to the N terminus of HBV miniP (S5A Fig).

In BL21* Codonplus (Cp) cells products of the expected ~100 kDa molecular mass were massively expressed yet nearly insoluble (S5B Fig); amongst other *E*. *coli* strains tested only Arctic Express (DE3) cells (Agilent), induced at 12°C, generated a Coomassie Blue detectable fraction of the protein in soluble form (*see below*). To enrich the HBV miniP protein from the BL21*CP cell inclusion bodies we employed an analogous protocol (S5C Fig) as previously established for DHBV miniP [26]. In brief, HBV miniP in inclusion bodies was solubilized in 7 M guanidinium hydrochloride (GuHCl), then rapidly diluted into excess cold renaturing buffer containing 1.5 M non-detergent sulfobetain (NDSB 201) as main aggregation-preventing ingredient [60]; after one hour on ice remaining insoluble components were removed by centrifugation and the supernatant was used for subsequent experiments.

There we subjected the renatured HBV miniP to conditions established for DHBV miniP [26], namely RNP formation with ε1 RNA (providing a final ~1 mM Mg^2+^ concentration from in vitro transcription), and subsequent supplementation with Mn^2+^ (to 2.5 mM final concentration) and α^32^P-dTTP for priming. This yielded a robust signal of ^32^P-labeled P protein at the expected position (Fig 4B). RNAs ε2 to ε5 produced similar albeit weaker signals which were not seen with ε wt and the single-site variant ε0, and neither with Dε RNA, tRNA or no RNA (Fig 4B, *right panel*). Signals from RNA variants εn1 and εn2 which had exerted lower replication competence (Fig 2B) were barely detectable.

Together these data demonstrated the feasibility of setting up a simple HBV in vitro priming system using renatured HBV miniP protein plus appropriately modified ε RNAs as the only macromolecular components.

### Two basepair-reducing mutations in the upper ε stem suffice for in vitro priming activity

**Fig 5:**
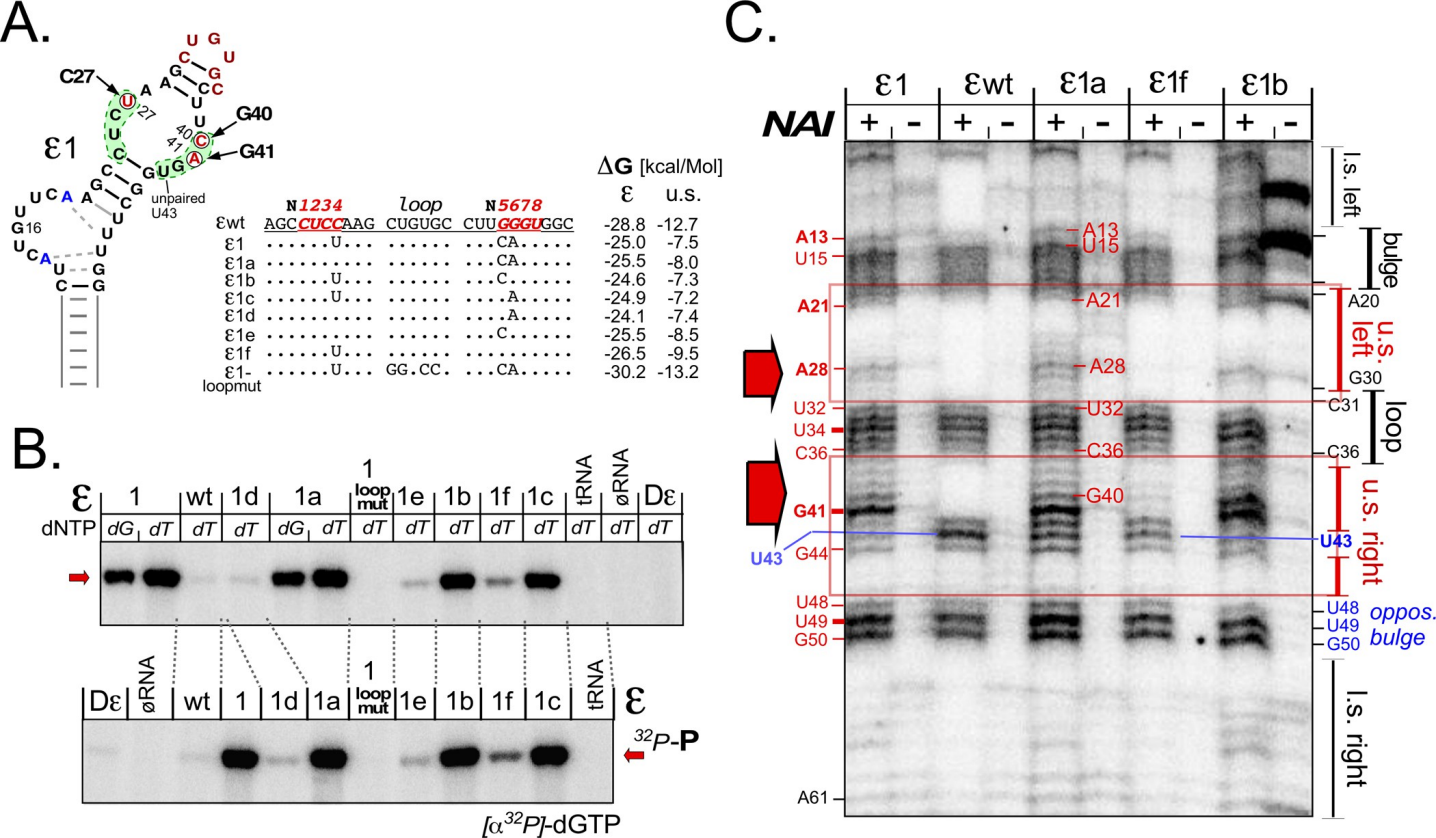
Two upper stem mutations are necessary and sufficient for robust in vitro priming with renatured HBV miniP. **(A) Scheme of secondary mutations introduced into variant ε1.** To further approximate ε1 to wt ε its three mutations were individually and in combination reverted, yielding variants maintaining two (ε1a,b,c) or only one mutation (ε1d,e,f); in addition, four loop mutations preventing pgRNA packaging in cells were introduced into ε1 context (ε1-*loopmut*). ΔG values were derived as in Fig 2. **(B) In vitro priming activities with renatured HBV miniP.** Assays with the indicated RNAs were performed as in Fig 4B, using α^32^P-dTTP (*top*; except in lanes labeled dG) and α^32^P-dGTP *(bottom*). The double mutants performed similarly well with either dNTP while signals from the single-site mutants were much weaker and the loop mutant gave no signal at all. **(C) Robust priming activity correlates with a loosened upper stem.** The indicated RNAs were analyzed by SHAPE at 37°C as in Fig 3. Highly priming active variants ε1, ε1a and ε1b showed extra signals around A28 and between C36 and G41 (*red arrows*), with the G41 signal exceeding that from U43, indicative of an ε1-like open upper stem structure. The band pattern of the poorly priming variant ε1f essentially conformed to that from wt ε.

The best-performing RNA, ε1, differs at three positions from ε wt. To test whether fewer mutations suffice for in vitro priming activity we reverted the mutations in ε1 back to wt, individually and in all combinations. RNAs maintaining two mutations were termed ε1a-c, and the single-site mutants ε1d-f (Fig 5A). In priming assays with renatured HBV miniP and α^32^P-dTTP (lanes marked dT) all double-mutants generated similarly intense signals as the parental ε1 RNA, while the Dε, tRNA and no RNA controls remained negative (Fig 5B, *top panel*); notably, ε1 and ε1a produced comparably strong signals with α^32^P-dGTP (lanes marked dG), in line with A- and C-templated reactions (see below). Signals with the single-site mutants ε1d, ε1e, ε1f were much weaker. Here a very faint band was seen with ε wt RNA whereas the lane with a further control RNA, ε1a-*loop_mut*, remained completely blank; the respective loop mutations had blocked pgRNA encapsidation in cells [13]. Testing the other ε RNAs including wt ε for α^32^P-dGTP priming (Fig 5B, *bottom panel*) revealed no major differences to α^32^P-dTTP priming.

To verify a correlation between in vitro priming activity and upper ε stem destabilization we probed the structures of the active variants ε1a and ε1b versus that of poorly active ε1f by SHAPE; ε1 and ε wt were included for reference and as reproducibility controls. The band patterns were indeed superimposable with the previous SHAPE analyses, with common signals at the loop, the bulge, around the unpaired U43 and at U48-G50 opposite the bulge. Again ε1 generated extra bands around A28 in the upper left stem, and from C37 to G41 in the upper right stem (Fig 5C, red arrows), with G41 rather than U43 as in ε wt as the strongest signal in this region. As already evident by visual inspection, the same distinct features were present in RNAs ε1a and ε1b (though somewhat obscured by probably degradation-related bands in the -NAI lane; *far right*) but absent from ε1f which presented like ε wt. Hence in vitro priming activity correlated with accessibility of the residues in the center of the upper stem.

To independently confirm the importance of structural flexibility for ε function we examined ε variants with deliberately stabilized ε upper stems. The first set comprised Dε and two chimeras (S6A Fig) in which the Dε upper stem was replaced by that of either HBV ε wt (D/Hεwt) or ε1 (D/Hε1); Dε´s upper stem is rather tolerant towards mutations as long as the apical GU-rich motif f is maintained [30]. In vitro priming with renatured DHBV miniP (miniDP) and α^32^P-dGTP yielded strong signals with wt Dε RNA, a weaker signal with D/Hε1, and only a marginal signal with D/Hεwt. When introduced into the wt DHBV expression vector pCD16 [40], D/Hε1 supported substantial replication whereas D/Hεwt did not (S6B Fig).

We also introduced stabilizing mutations into the HBV 5´ ε sequence in vector pCH-9/3091 (S6C Fig), namely U25C which converts a U-G to a more stable C-G pair, ΔU43 which removes the unpaired U and creates a contiguous double-helix, and the corresponding double mutant U25C_ΔU43, boosting predicted ΔG values from -28.8 (ε wt) over -31.8 (U25C) and -32.6 ΔU43 to -35.6 kcal/Mol (U25C_ΔU43). When transfected into Huh7 cells in parallel with the wt vector and its ε1a derivative all generated comparable amounts of capsids while Southern blotting revealed the typical viral DNA signals only in the wt, ε1a and U25C samples, with decreasing intensities; no capsid-associated DNA was seen for mutants ΔU43, in line with previous data [14], and U25C_ΔU43 (S6D Fig). Hence also in cells can an overly stable upper stem impair a productive P-ε interaction, although the lower replication level of the ε1a vs. the ε wt construct indicates that additional factors affect replication performance.

### Two major initiation sites in the 3´ proximal bulge region

**Fig 6:**
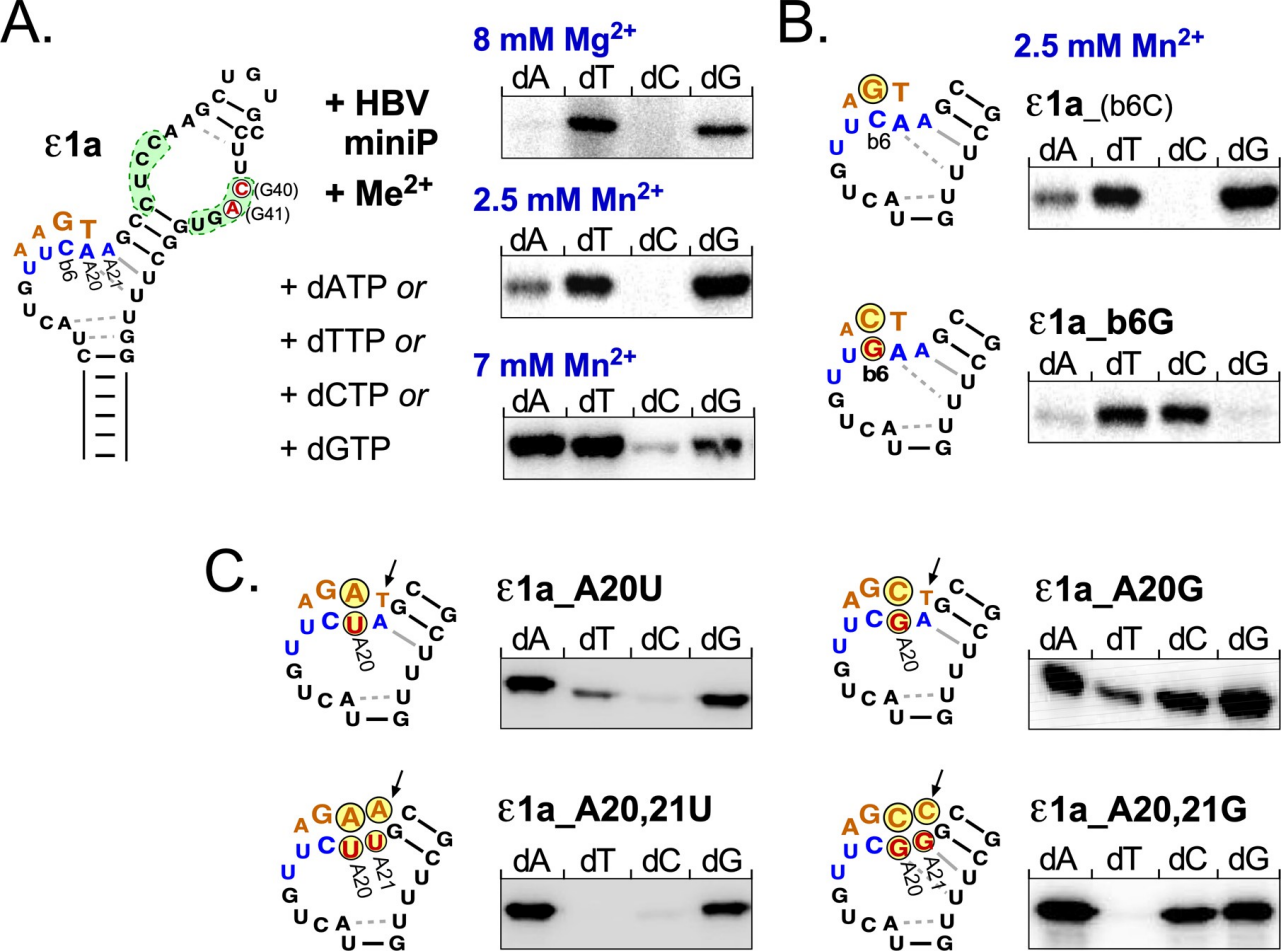
ε1a RNA directs site-specific replication initiation in vitro. **(A) Impact of bivalent metal ions on dNTP utilization.** In vitro transcribed ε1a RNA was subjected to priming reactions with renatured HBV miniP in the presence of the indicated Me^2+^ concentrations and one each of the four natural dNTPs. The presumed template region around classical bulge position 6 (b6) and A20 (not paired according to SHAPE) and A21 plus the templated DNA nt are indicated in blue and red lettering in the structure model (*left)*. Mg^2+^ concentrations ≤2.5 mM yielded very weak signals; at 8 mM Mg^2+^ dTTP and dGTP produced clearly detectable signals (*top*). However. signal intensities were much enhanced by Mn^2+^, at 2.5 mM revealing low level priming also with dATP but not dCTP (*middle*); at 7 mM Mn^2+^ dATP priming was much enhanced, dGTP priming somewhat reduced, and dCTP priming gave a well detectable band (*bottom*). Hence subsequent assays were routinely performed at 2.5mM Mn^2+^. **(B) Bulge position 6 directs dGTP utilization.** The C>G mutation at b6 (ε1a_b6G) switched dNTP preference from dGTP to dCTP but had little impact on dTTP and dATP priming. The autoradiogram for wildtype ε1a (termed here ε1a_(b6C) to emphasize the nt at b6) is the same as in the middle panel of A. **(C) Bulge-following nucleotides A20, and to some extent A21, can direct dTTP utilization.** Efficient dTTP utilization in all assays suggested A20 and possibly A21 as templates. Mutation A20U reduced dTTP priming and enhanced dATP priming (*top left*). Double mutation A20,21U virtually abolished dTTP priming (*bottom left*). Mutations A20G *(top right*) and A20,21G (*bottom right*) gave analogous results regarding dTTP priming but additionally enabled dCTP utilization.

The data described above demonstrated a robust priming activity of HBV miniP with select ε RNAs and either dTTP or dGTP, suggesting as templates the 3´ proximal C of the bulge ("b6") or the following A20, which according to the SHAPE data (Figs 5 and S4) even in wt ε is not paired as usually drawn. For confirmation we subjected renatured HBV miniP and ε1a RNA to different in vitro priming conditions; these included assessing a potential impact of Mn^2+^ on nt specificity of the priming reaction [48], although our previous DHBV data supported enhanced but not less specific in vitro priming with 2.5 mM Mn^2+^ compared to Mg^2+^ [26]. In pilot experiments we saw no priming signals with 2.5 mM Mg^2+^ for any of the four dNTPs; however, at 8 mM Mg2+ an overnight phosphoimager exposure revealed specific signals with dTTP and dGTP but not dATP or dCTP (Fig 6B, *top panel*). In a parallel priming reaction supplemented with 2.5 mM Mn^2+^, phosphoimager signals were already evident after 1–2 h exposure; again dTTP and dGTP gave the strongest signals, while a weak signal became detectable with dATP but not with dCTP. At 7 mM Mn^2+^, the signal with dATP increased substantially, that with dGTP decreased, and now also dCTP produced a detectable though very faint signal. Hence 7 mM Mn^2+^ may indeed affect nt specificity whereas 2.5 mM Mn^2+^ yielded similar data to 8 mM Mg^2+^ but at a higher level and was then used throughout. To confirm the template function of the C residue at b6, we replaced this nt in ε1a by G (ε1a_b6G) which should mediate dC incorporation; exactly this was observed (Fig 6B, *lower panel*). To corroborate the assumed template function of A20 for dT incorporation we extended the assays to mutant ε1a_A20U. The mutation enhanced dATP priming and markedly weakened dTTP priming but did not ablate it (Fig 6C, *top left panel*). However, when the following A21 was also changed to U (ε1a_A20,21U) the dT signal vanished (Fig 6C, *bottom left panel*), compatible with A20, and to a low extent also A21, being usable as template. dCTP had given only marginal or no signals in the assays so far, in line with the absence of a G residue within the template region defined above. In contrast, variant ε1a_A20G used dCTP rather efficiently while dTTP utilization was reduced (Fig 6C, *top right panel*). Analogously to ε1a_A20,21U, the double mutant ε1a_A20,21G showed no more dTTP priming at all (Fig 6C, *bottom right panel*).

Hence the two major templating sites for HBV priming, in vitro, are the last nt of the classical bulge, b6, and the following A20, formerly considered as part of the upper stem but by our SHAPE data largely unpaired. As seen in Fig 1B, in HBV trimeric 5´-GAA primers as well as tetrameric 5´-TGAA primers fully match the acceptor site at DR1*. In support of the replication relevance of the in vitro priming data transfected HBV genomes carrying the ε1 and the ε1a sequence produced (-)-strand DNAs whose 5´ ends matched those from wt HBV, as shown by primer extension (S7 Fig). Hence within the method-intrinsic uncertainty of 1 or 2 nt ε1 and ε1a are valid models for wt ε.

### A properly split ε RNA enables in vitro priming with the wt ε sequence

**Fig 7:**
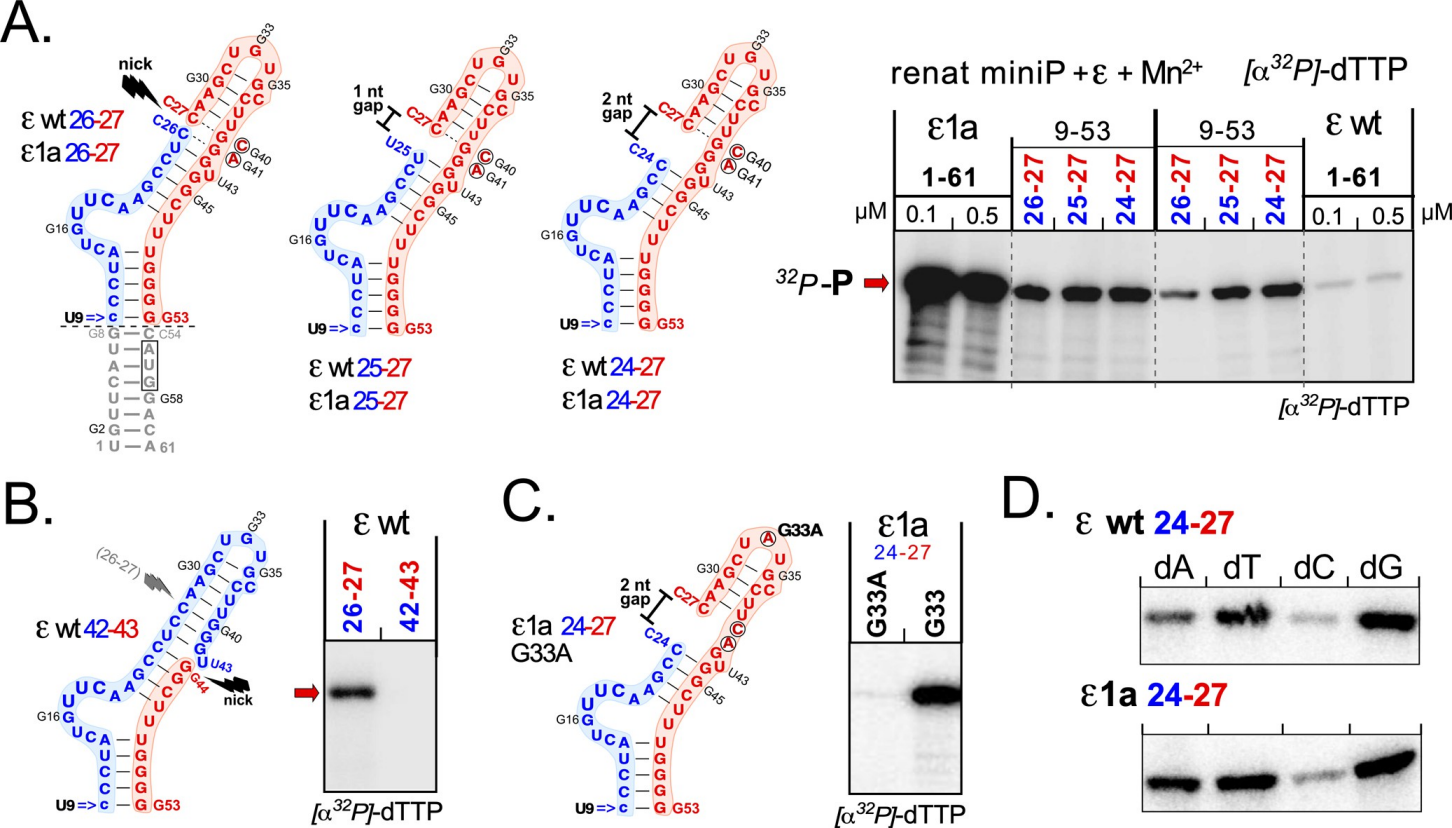
Bipartite ε RNAs enable in vitro priming in the absence of destabilizing ε1a mutations. **(A) Synthetic split ε RNAs.** Omitting the bottom half of the lower stem (nt U1-G8 and C54-A61, i.e. retaining the sequence 9–53; grey lettering) two kinds of chemically synthesized RNAs were annealed. The 5´ proximal oligos (blue) spanned nt 9 (changed from U>c for a more stable c9-G53 pair) to positions C26, U25, or C24; the 3´proximal oligos (red*)* comprised nt C27-G53, either with the wt G residues at positions 40 and 41, or with the G40C plus G41A mutations from ε1a. Annealing of the oligos ending at position 26 and starting at 27 should give ε wt and ε1a stem-loops with a nick; oligos ending at positions 25 and 24 would create one and two nt gaps (*middle and right*). The indicated annealed oligos (at 0.5 μM final concentration), and for comparison in vitro transcripts comprising the contiguous full-length stem-loops ε1a and ε wt (at 0.1 and 0.5 mM), were subjected to in vitro priming with α^32^P-dTTP and renatured HBV miniP. **(B) The split position affects in vitro priming template activity.** Two RNA oligos spanning ε positions 9–42 and G43-G53 were annealed (ε wt 42–43) and used for dTTP priming as in A, in parallel to ε wt 26–27 as in A. **(C) A pgRNA packaging preventing loop mutation (G33A) inhibits in vitro priming activity of gapped ε1a RNA.** RNA stem-loop ε1a 24–27 and its G33A homolog were examined side-by-side by in vitro dTTP priming. **(D) Gapped ε stem-loops display comparable dNTP preferences as contiguous in vitro transcripts.** The indicated gapped stem-loops were subjected to in vitro priming with renatured HBV miniP and each of the four natural dNTPs, as in Fig 5B. Both synthetic stem-loops gave the strongest signals with dTTP and dGTP, followed by dATP and finally dCTP.

As a mutation-independent approach for validating the importance of structural flexibility we introduced nicks and gaps into the upper ε stem, practically by annealing two chemically synthesized RNA oligos into a "split" stem-loop (Fig 7A). To minimize synthetic cost, we shortened the lower ε stem to the five classical top basepairs, and replaced the authentic U9-G53 pair by a more stable c9-G53 pair (constructs 9–53). To facilitate adoption of the ε-typical structure we placed the split site between residues 26 and 27 in the center of the upper left half-stem (Fig 7A), creating split ε RNAs wt 26–27 and ε1a 26–27. In addition, we used upstream oligos lacking one or two 3´ terminal nt, yielding split RNAs ε wt 25–27 and ε1a 25–27, and ε wt 24–27 and ε1a 24–27 with 1 nt or 2 nt gaps in the annealed structure (Fig 7A). We then subjected the synthetic RNAs, and in parallel in vitro transcribed 61 nt ε wt and ε1a RNA, to the priming assay conditions described above with renatured HBV miniP protein. As before, the contiguous ε1a RNA gave a strong signal, that with ε wt remained marginal (Fig 7A, *right*). However, distinct signals were also produced by both split RNAs, demonstrating a clear enhancement by the discontinuity in the wt ε sequence. Whether the weaker signal with the nicked vs. gapped wt ε RNAs originates from a stronger destabilization in the gapped variants remains to be determined. However, that the reactions with the split ε RNAs reflect features of authentic HBV protein-priming is supported by additional experiments. As shown in Fig 7B, an analogous split in the right half-stem of wt ε (ε wt 42–43) did not support priming, perhaps owing to particular features in an alternative, priming-active RNP structure (see below). More directly, a single mutation in the loop, G33A, which inhibits pgRNA packaging in cells (mutant Loop3 in [14]), prevented in vitro dTTP priming in the split ε1a 24–27 context (Fig 7C). Moreover, in priming assays with different dNTPs, the gapped ε1a as well as wt ε RNAs displayed a comparable preference for dGTP and dTTP, and to some extent dATP, but not dCTP as contiguous ε1a RNA (Fig 7D). Hence destabilizing the upper ε stem by disrupting the RNA backbone at an appropriate site similarly enabled in vitro priming as reducing the local basepairing potential by mutations. Furthermore, the successful employment of synthetic RNAs as priming substrates opens new avenues for structure-function studies that are no more limited to the four natural NTPs.

### A solid-phase HBV in vitro priming system reveals a distinct conformational change in ε1a RNA upon P protein binding

**Fig 8:**
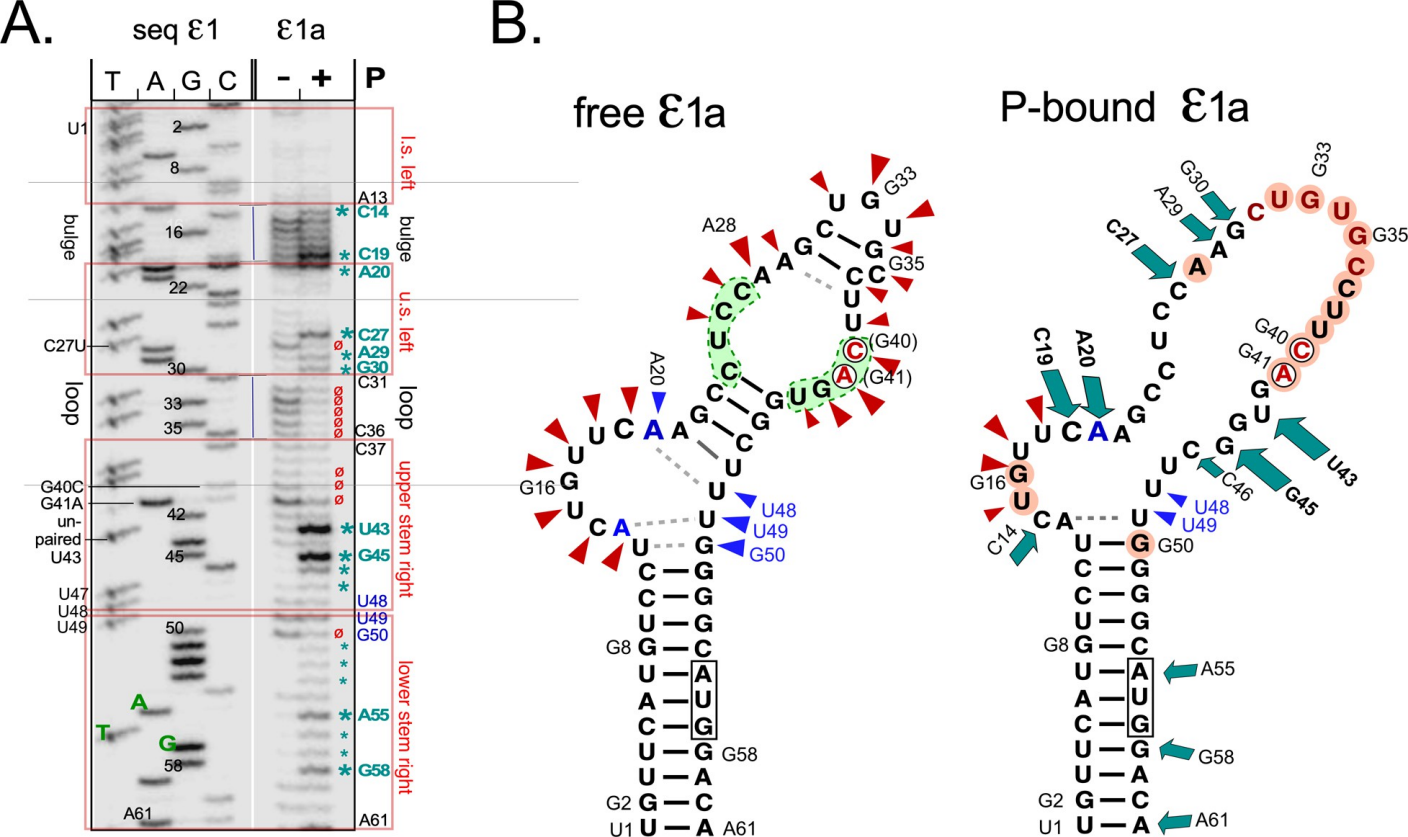
A major conformational shift in ε1a RNA upon P binding revealed by natively purified immobilized miniP. Biotinylated MBP-HBVminiP-BAP expressed at 12°C in *E*. *coli* Arctic Express cells and present in the soluble fraction of the cell lysate was bound to monomeric avidin beads (S8 Fig), washed and incubated with in vitro transcribed ε1a RNA. After 1 h at 30°C, the bead-bound RNA was reacted with NAI and released from the beads by boiling in SDS sample buffer for primer extension as in Fig 5. In parallel, free ε1a was subjected to SHAPE analysis**. (A) Denaturing PAGE analysis of extension products from free versus P-bound ε1a RNA.** Bands with enhanced reactivity in the bound state are indicated by green asterisks, those with reduced reactivity by red ø. **(B) SHAPE hits superimposed on secondary structure models of free and P-bound ε1a RNA.** Data for free ε1a fully reproduced those shown in Fig 5; positions with enhanced NAI accessibility in bound RNA are highlighted by green arrows, positions with lower reactivity than in free RNA by light red background. Slightly enhanced signals in the lower right stem will require further investigation.

When exploring different options for HBV P protein expression in bacteria we also examined an MBP-miniP protein carrying a C terminal Biotin-Acceptor-Peptide (BAP; also known as Avitag [61] and comprising the aa sequence GLNDIFEAQKIEWHE), termed MBP-HBVminiP-BAP (S8A Fig); in *E*. *coli*, the central K residue becomes biotinylated by the endogenous *E*. *coli* BirA biotin ligase, enabling various avidin-based affinity procedures, including detection by peroxidase-conjugated streptavidin (S8A Fig). The protein was virtually insoluble when expressed in BL21*Cp cells or SHuffle T7 Express cells at 20°C but remained partly soluble in ArcticExpress (DE3) cells (Agilent) at 12°C; these cells produce large amounts of the GroEL/GroES analogous cold-shock chaperonins Cpn60/Cpn10, supposed to facilitate folding and reduce aggregation of heterologous proteins. As protein accumulation was slow (S8B Fig, *right panel*) cultures were routinely kept under inducing conditions for 24 h before harvest. As shown in S8C Fig the BAP-tag enabled immobilization of the HBV miniP protein to Monomeric Avidin agarose beads (Pierce), from which 2 mM D-biotin eluted small amounts of an ~100 kDa protein plus an excess of the 60 kDa Cpn60 chaperonin. When an aliquot of the beads was directly boiled in SDS sample buffer the 100 kDa band was detected at a higher ratio to Cpn60, together with numerous but weak contaminant bands (S8C Fig). Although this suggested that at least part of HBV miniP did not behave like a typical soluble protein, comparison with the input sample showed a marked enrichment on the avidin beads. To test for priming activity we repeated the avidin enrichment but omitted the D-biotin elution step, then added to equal aliquots of the beads RNAs ε1a, ε wt, or Dε, or tRNA or no RNA in Mg^2+^ containing buffer to allow RNP formation, then supplemented the reactions with Mn^2+^ and ^32^P-dTTP. Potential on-bead priming products were released by boiling in SDS sample buffer and resolved by SDS-PAGE and subsequent autoradiography. Reassuringly, also this miniP preparation gave a well detectable signal with ε1a RNA but not the others and neither without RNA (S8C Fig). Hence ε1a RNA was capable of productive RNP formation with the bead-bound HBV miniP protein, providing an opportunity to monitor potential structural changes between free and bound ε1a RNA. To this end, ε1a RNA was subjected to SHAPE analysis as before, or reacted with NAI after a one hour binding step to immobilized HBV miniP. Both reactions were then analyzed side-by-side (Fig 8A). Consistent with the previous data (Fig 5C) free ε1a RNA showed accessible regions at the bulge (plus unpaired A20), the loop, around A28 and G41, and opposite the bulge (U49, G50). The pattern of the bound ε1a RNA was remarkably different, with various positions producing enhanced (green asterisks in Fig 8A) and others reduced signal intensities (red ø symbols). The bulge still generated pronounced signals, yet those from the template nt C19 and A20 were strikingly enhanced. All loop signals were reduced, including the following U39 and the mutated G40C and G41A positions, as well as G50 opposite the bulge. The most prominent signal increases beyond C19 and A20 were in the left half stem from C27 to C31 (though not A28), and U43, G45 and C46 which precede U48—G50 opposite the bulge. Enhanced signals were also seen in the lower stem at A58, the initiating A of the core ORF and G58, the fourth nt in the ORF. The differences in nt accessibility in free vs. P-bound ε1a RNA are schematically shown in Fig 8B. Exposure of residues in the right half-stem plus the reduced signals in the classical loop region are remarkably similar to the changes seen in DHBV P-bound Dε RNA [39]. While reduced signals could result from new intra-RNA as well as RNA-protein interactions, enhanced signals clearly indicate increased accessibility. Hence in sum these data provide proof-of-principle for a major conformational rearrangement in HBV ε RNA upon complex formation with HBV P protein, akin to the upper stem opening in Dε. Our new in vitro priming systems provide suitable tools for future detailed mechanistic examination.

## Discussion

For DHBV, in vitro reconstitution of protein-primed replication initiation is long established while for human HBV the closest approximation is the isolation of preformed P protein—ε RNA complexes from transfected mammalian cells [45,46]. As one possible explanation we here focused on the higher stability of the upper stem in HBV ε which might impair, in vitro, a DHBV-analogous rearrangement of this region into a new priming-competent conformation that ensures proper positioning of the RNA template, the complementary dNTP, and the TP tyrosyl acceptor in the active site of P protein´s RT domain [26]. Hence reducing stability of the HBV ε upper stem might overcome this obstacle even without cellular factors and/or energy.

Employing an in-cell SELEX scheme we identified several functional non-wt ε sequences, including some with less stably folded upper stems. As hypothesized, several of these RNAs supported in vitro priming by HBV P protein in RRL, yet also in a simplified format requiring only truncated HBV miniP protein plus the modified ε RNA stem-loop as macromolecular components. Two upper stem mutations were sufficient for this activity, and their local structure-relaxing impact was demonstrated by SHAPE structure probing. For wt ε this revealed, in addition, a more open structure of the entire bulge region than previously assumed. In vitro priming was ε RNA-dependent and ε-templated, with the 3´ terminal C residue of the bulge and the following A residue as the major initiation sites. A first glimpse into the opportunities offered by this new in vitro system is our demonstration of a distinct conformation change in HBV ε1a RNA upon P protein binding; many more applications can be envisaged, in particular with the recent discovery of the HBV-related fish nackednaviruses [59] which despite their huge evolutionary distance employ a fundamentally similar mechanism of protein-primed reverse transcription [15].

### Restricted conformational dynamics impede HBV in vitro priming

Cell-free DHBV systems provided strong evidence that Dε-dependent replication initiation at a defined sites involves a rearrangement of the apical Dε region [29,30,39] that puts all components in place. The classic notion that the bulge, created by stable lower and upper stems, provides such positional information by defining its 3´ terminus as a privileged template position is likely an oversimplification. For instance, RNA transcription from dsDNA templates typically involves opening of 13 nt around the initiation site to create a transcription bubble [62]; hence for HBV ε a "bubble" of only 6 nt with the 3´ terminal position fixed in space by the upper stem seems unlikely. The ease of generating a more open RNA structure in the RNP complex depends on the free energy balance between loosing favorable intra-RNA interactions versus gaining new RNA—P protein interactions. These latter interactions are energetically poorly characterized, but the much lower stability of the upper stem in Dε vs. HBV ε is obvious [38,39]; hence we reasoned that for Dε the interaction with P protein *per se* is sufficient for in vitro restructuring whereas for HBV ε auxiliary factors and/or energy or, as pursued here, an artificially diminished RNA restructuring cost are required.

The classical representation of hepadnaviral ε elements as bipartite stem-loops has shaped the perception that both stems are crucial for function. However, this is only well-documented for the lower stem [29,30,42,63]. The upper stems in all non-duck avihepadnavirus ε elements are less stably folded than in Dε [29], and even Dε function, in vitro and in cells, is not dependent on such stability [30]. Moreover, stable lower but not upper stems are found in nackednavirus ε elements, including those with demonstrated in vitro priming activity [15].

From a library of HBV genomes with partially randomized ε upper stems we were able to identify some 20 replication-competent non-wild-type sequences (Figs 2 and S2 and S3), including some with predicted lower upper stem stability. SHAPE analyses confirmed such localized destabilization for the three-site variant ε1 and its derivatives maintaining two (ε1a,b,c) but not only one mutation (ε1d,e,f). Notably, the high resolution of SHAPE (S4 Fig) also revealed that in wt ε the region opposite the bulge (U48-G50) is much less constrained than implied by the classical 2D models, in consequence extending the bulge (Figs 3, 5, and S4).

Most importantly, several of the selected RNAs, prominently ε1 and ε1a, ε1b and ε1c, generated well detectable priming signals with in vitro translated full-length HBV P (Fig 4A) yet also with bacterially expressed HBV miniP protein (Fig 4B). ε RNA dependence and specificity is documented by the lack of signals with tRNA or Dε RNA, or without RNA (Figs 4 and 5). The only marginal signals with wt ε strengthen our hypothesis that too stable basepairing underlies its inability to support in vitro priming. Stability of the upper stem may become limiting even in cells, as suggested by the reduced or ablated replication capacity of increasingly stabilized DHBV/HBV ε chimeras (D/Hε1, D/Hεwt) and HBV ε point mutants U25C, ΔU43, or their combination (S6 Fig). As hypothesized, all in vitro priming active RNAs had lower predicted stability than wt ε; however, that the even less stable variants ε3, ε4, and ε5 gave weaker signals than ε1 (Fig 4) suggests sequence-specific contributions, perhaps due to less favorable P protein interactions in the active complex.

At any rate did the SHAPE data (Fig 6) reveal a largely closed upper stem in the priming-inactive wt and ε1f RNAs, versus a centrally open structure in the priming-active ε1, ε1a and ε1b RNAs. From there the mostly unpaired upper stem in the P-bound state (Fig 8) is likely much easier to access. Notably, SHAPE at 60°C revealed no local melting in wt ε (S4 Fig), in accord with biophysical data indicating a melting temperature of 79.5°C in 150 mM NaCl [38] and thus a high resilience against conformational alterations.

Altogether these data fully support the hypothesis that the high stability of the upper stem in HBV wt ε prevents it from adopting a new, priming-active conformation in vitro.

### Template-specific initiation site selection in vitro

Conceptually, ε-templated 3 to 4 nt long DNA oligos initiated at C19 (5´-GAA) as well as A20 (5´-TGAA; Fig 1B) fully match the DR1* acceptor site, with experimental support for both [19,45]. Our initial in vitro priming assays using ε1 and ε1a RNA gave comparably strong signals with dTTP and dGTP (Figs 4, 5 and 6). These data would also fit to an indiscriminate use of any dNTP. Our analysis of Me^2+^ dependence suggested indeed a more indiscriminate use of dNTPs (in particular dATP) at high Mn^2+^ concentration but also confirmed the stimulatory effect of 2.5 mM Mn^2+^ on priming efficiency without compromised nt specificity. Utilization of dTTP and dGTP was comparable under all conditions, while signals with dCTP always remained marginal (Fig 6A), consistent with the lack of any nearby G templates. Conversely, replacing b6C by G shifted dGTP utilization to dCTP (Fig 6B), indicating this residue as one of two major initiation sites. The nearly unchanged intensity of the dTTP signal (Fig 6B) suggested that the following A20 can also act as template, as supported by the near loss of dTTP signal but gain of dATP signal in variant ε1A_A20U, and of dCTP signal in variant ε1a_A20G (Fig 6C). Hence dNTP choice correlated strictly with nt identity at the 3´ terminus of the classical bulge structure (b6) and the following A20, usually drawn as bottom basepair of the upper stem but as accessible as b6 by SHAPE analysis and super-exposed in the P-bound state (Fig 8). Notably, the weak remaining dTTP priming signals were completely ablated when A21 was also changed to U or G (Fig 6C, *bottom panels*); hence A21 may also, yet inefficiently, act as priming template.

Overall, these data provide a coherent picture of initiation site selection whereby mainly two nucleotides 6 to 7 positions downstream the start of the bulge, as defined by the stable lower stem, act as priming templates. By necessity, these data were obtained using mutant ε RNAs; however, their virological relevance is supported by several lines of evidence. First, the in vitro priming enabling mutations in ε1 and ε1a did not evoke the production of aberrant ε-templated primers in cells, as the 5´ residues of the respective (-)-DNAs were highly similar to those from a wt ε HBV genome (S7 Fig). Second, synthetic split ε RNAs with a nick, or a one or two nt gap in the left upper half-stem enabled in vitro priming even if based on the wt ε sequence (Fig 7); moreover, dNTP preference was the same as with the contiguous transcripts, and a point mutation in the loop (Fig 7C) that prevents pgRNA packaging in cells [14] also prevented in vitro priming activity; in contrast, non-productive in vitro HBV P protein binding occurs even if the entire loop is deleted [42]. In the absence of more detailed structural knowledge we ascribed the activity of the split ε RNAs largely to the many more degrees of conformational freedom around the "split" phospho-diester bond compared to the contiguous chain where the covalent linkage favors maintenance of a continuous double-helix. In addition, breathing of the artificially created new terminal basepairs would make the nicking site more flexible and reduce resilience of the entire structure against restructuring. Not the least did the SHAPE data also for wt ε demonstrate a high accessibility of U48 and especially U49-G50 (Figs 3, 5C and S4B) opposite the bulge. All these residues are classically drawn as paired, U48 with A20 forming the top closing pair of the bulge, and U49 and G50 with A13 and U12, respectively, the bottom basepairs that define the bulge (Fig 1B). A13 and A20 gave well detectable signals as well, suggesting the bulge to extend by at least one nt into the classical upper and lower stems. Besides resembling Dε with its unpaired U opposite the bulge (Fig 1B) these data are also fully compatible with our initiation site mapping.

Hence although the mutations in ε1 RNA and its derivatives are necessary for in vitro priming these RNAs are valid HBV ε models, only with more easily restructured upper stems.

The functional relevance of such restructuring was in particular supported by the DHBV P protein-mediated opening of the upper stem in priming-competent Dε RNAs [39]. Key changes, detected by Pb^2+^ probing [64], were a reduced accessibility from the apical loop through the entire right upper half-stem; however, a GUG motif in its center became hyperexposed. A solid-phase format of the HBV miniP—ε1a RNA interaction assay demonstrated very similar alterations in the P-bound HBV RNA. Residues from the loop to G50 at the top of the lower stem became protected while U43 and G45, slightly downstream the center of the right half-stem became overexposed. Notably, strongly enhanced signals were also seen for C19 (i.e. b6C) and the following A20 near the 3´ end of the bulge, i.e exactly the two mapped major initiation sites. Hence the RNA conformations in priming-active DHBV and HBV ε—P protein complexes appear principally similar, even though wt HBV ε and Dε do not support cross-priming by the heterologous P protein. Notably, however, we recently found that a few RNA mutations suffice to alter P protein specificity [15].

We therefore propose that the model outlined in Fig 1B for DHBV also holds for HBV, except that the higher RNA restructuring cost requires additional resources which are apparently only available in live cells. Candidates are cellular factors that co-enrich with affinity-tagged P protein—ε complexes in transfected cells [42], yet also other RNA unwinding or chaperoning activities, e.g. from the translation machinery, cellular ε binding or modifying [65] small molecules, or even the general ionic, temperature and/or energy conditions in a live cell. Given transfected HEK 293 cells support formation of active P—ε complexes in the absence of core protein [46], a proposed RNA chaperoning by HBc [66] may become relevant at later replication steps.

Why HBV has evolved a higher barrier than avihepadnaviruses between a stably folded and a more open RNA conformation associated with priming-competence remains to be resolved. One option is protection by the high local stability of the ε fold against nonproductive basepairing with other regions on pgRNA or cellular RNAs. Another, dynamic view relates to the transient nature of the priming-active RNA conformation which is required at just one specific step of replication but must give way to consecutive alternative conformations when the ε-templated primer is elongated, released and transferred to DR1*, or when the ε sequence is copied during minus-strand DNA synthesis or serves as template during pgRNA translation. In the latter setting, the stable ε stem-loop could confine translation efficiency to a level that does not interfere with polymerase binding and its replicative functions. Similarly, a stable ε stem-loop warrants that structure-dependent features, e.g. the nucleotides from the bulge and the loop, remain exposed until needed, enabling their interactions with polymerase or cellular partners. After such interactions, a high energy barrier between the different states ensures a strict distinction and therefore tight regulation. More specifically, opening of the upper stem upon polymerase binding (Fig 8) is compatible with facilitated basepairing of ε with the ϕ element. ϕ is a 19 nt sequence upstream 3´ DR1* with 17 nt complementarity to the left half-stem of ε, excluding the bulge [67–69]. Mutations reducing the ε-ϕ basepairing also reduced minus-strand DNA synthesis, likely by disfavoring transfer of the 5´ ε-templated primer to the 3´proximal acceptor (Fig 1B). Several of our ε variants bearing mutations in the N1-N4 positions (εn1, εn2, ε3), part of the proposed ε-ϕ interaction region, appeared to have indeed lower replication capacity; this might have contributed to the enrichment of wt genomes in our in-cell SELEX experiments (S2 Fig). The less obvious replication deficiencies of mutants ε4 and ε5 (Figs 2B and S3) may relate to the mutations in the right half-stem which weaken only the intra-ε basepairing, thus restoring an adequate energy balance between the alternative structures. However, other scenarios are conceivable and further systematic data are required for firm conclusions. Notably, the transitions between free and polymerase- and/or ϕ-bound ε likely reflect only part of the pgRNA dynamics. Classic precedents are the conformational changes during the lifecycle of RNA viruses such as bacteriophage MS2 or in hepatitis delta virus (HDV) ribozyme activation [67], yet many more RNA based regulatory mechanisms are emerging [68]. Such diversity leaves ample room even for related viruses such as HBV and DHBV to employ nonidentical solutions to meet a common demand, and it also suggests that HBV RNA offers even more new therapeutic targets than the ε-polymerase interaction.

### Conceivable practical improvements and new experimental opportunities

Our data support the validity of using structurally destabilized ε RNAs to model human HBV replication initiation in vitro, either via mutational reduction of basepairing, or by physical interruption of the contiguous RNA chain in the left upper half-stem. A foreseeable advance on the RNA side is to incorporate non-natural nucleotides for biochemical (e.g. cross-linking, affinity tagging) and biophysical applications (e.g. isotope labeling for NMR) aiming to understand the P—ε interaction in molecular detail. The major current limitation is the production of functional HBV P protein in larger quantities. In vitro translation in RRL yields active protein but only in the low microgram range. The HBV miniP fusion protein was highly expressed in bacteria (several milligram per 200 ml of culture) but, depending on expression strain and conditions, was virtually insoluble. That the same renaturation protocol as for DHBV miniP [26] also yielded active HBV miniP indicates similar basic properties of the two enzymes. However, we do not know which fraction of the solubilized protein is enzymatically active. The avidin-bead enrichment of BAP-tagged miniP from Arctic Express cells has its own advantages, in particular the option to rapidly change conditions. The distinct SHAPE patterns for free vs. P-bound ε1a RNA indicate that at least part of the immobilized P protein molecules can specifically interact with the modified ε RNA although the exact fraction is again less clear. Revealing new basic science experiments could include a systematic comparison of the accessible residues in priming-active vs. inactive RNAs, in particular wt ε, and monitoring progression of the RNA structure from P protein binding through addition of the first and the subsequent nt to the ε-templated primer. High-throughput applications will, however, require improved P protein production methods. A promising option is a more easily upscalable in vitro translation system such as wheat germ extract (WGE) [70]. Though earlier reported as much inferior to RRL for production of active DHBV P protein [22] our recent data indicate WGE as a viable alternative for expression of P protein [15]. This could feasibly be combined with affinity tagging for solid-phase enrichment and thus form the basis of large-scale screening for protein-priming inhibitors as new and highly specific anti-HBV agents.

## Material and methods

### Cell lines and transfection

Culturing and transfection of the human hepatoma cell line Huh7 using Mirus LT1 transfection reagent were performed as previously described [71,72]. Absence of mycoplasma contamination was confirmed by a previously described PCR assay [73].

### Plasmid constructs

Plasmid pCH-9/3091 encoding a CMV-IE enhancer/promoter controlled HBV genome (genotype D, subtype ayw; Genbank accession no.: NC003977.2) and an analogous DHBV construct, pCD16, carrying a 1.1x DHBV16 genome (GenBank accession no. K01834) have previously been described [41,52]. Special constructs used in the in-cell SELEX procedure are detailed in S1 Fig.

For in vitro translation of HBV P protein in RRL the DHBV P ORF in vector pT7AMVDpol16H6 [63] was replaced by the complete HBV P ORF followed by a His_10_-tag, giving construct pT7AMVHpol_His10. Bacterial expression plasmids for HBV P protein derivatives were analogous to the T7 RNA polymerase controlled DHBV polymerase vector pET28-MBPminiP [26]; details are given in S5 and S8 Figs.

Constructs for in vitro transcription of HBV ε RNAs were derived from plasmid pUC19T7-Dε which carries between the Sal I and Eco RI sites of pUC19 a T7 RNA polymerase promoter followed by the Dε sequence [41]. The Dε part was replaced by HBV sequence 3124-3182/1-45 (ε stem-loop positions T3143-A7) followed by restriction sites for Hpa I and Cla I, and joined into the Eco RI site; linearization at the Eco RI site results in a nominally 120 nt long transcript (including three 5´ G residues from the T7 promoter). These plasmids were termed pUC19T7-HBVε. In some constructs, the HBV-specific sequence was terminated by mutation C12t creating a Cla I site (ATG GAC ATC GAt; Cla I site underlined) and immediately followed by the Eco RI site; here, linearization with Eco RI yields a nominally 79 nt long transcript; these plasmids were termed pUC19T7-HBVε_short. No substantial differences between the longer vs. shorter RNAs were observed in priming activity and/or secondary structure within the ε stem-loop part.

### Recombinant P protein expression

Full-length HBV P protein with a C terminal His-tag was generated by coupled in vitro transcription/translation of plasmid pT7AMVHpol_His10 in an RRL-based system (Promega) as recommended by the manufacturer. MBP-HBV-miniP-H6 was expressed from plasmid pET-MBP-HP1-199_292-601H6 in *E*. *coli* BL21*Cp cells and renatured from purified inclusion bodies as described in S5 Fig. MBP-HBV-miniP-BAP was expressed from plasmid pET-MBP-HP1-199_292-601-BAP in *E*. *coli* Arctic Express (AE) cells and immobilized to Monomeric Avidin Agarose beads (Pierce) as detailed in S8 Fig.

### RNA synthesis and structure probing

In vitro transcriptions were performed on the Eco RI linearized pUC19T7 plasmids using the AmpliScribe T7 high-yield transcription kit (Epicentre/Lucigen) as described [26]. Chemically synthesized RNA oligomers for split ε were obtained from biomers.net (Ulm, Germany). Enzymatic probing using RNases A (ThermoFisher), T2 (Invitrogen) and V1 (Ambion) was performed as previously described for Dε [39], using gel-purified 5´-^32^P-labeled in vitro transcripts.

SHAPE analyses were performed essentially as described [57,74]. In brief, 2-methylnicotinic acid imidazolide (NAI) was generated in situ by dropwise addition over 5 min of 162 mg (1 mMol) 1,1´-carbonyldiimidazole in 500 μl anhydrous DMSO to 137 mg (1 mMol) 2-methylnicotinic acid in 500 μl DMSO and further incubation for 1 h at room temperature. The resulting ~1 M NAI solution was used freshly, or stored at -80°C until further use. In a typical experiment, 3 μg RNA, obtained from in vitro transcription of the respective pUC19T7 plasmid cut with Pvu II (to generate longer 3´ ends than with Eco RI for the subsequent primer extension; see Fig 3B), in 10 μl 0.5x TE buffer (5 mM Tris-HCl [pH 7.4], 0.5 mM EDTA) was heated to 90°C for 2 min and flash-cooled on ice. After adding 5 μl 3x SHAPE buffer (333 mM HEPES, pH 8.0; 20 mM MgCl_2_, 333 mM NaCl) the RNA was allowed to renature for 20 min at 37°C. For acylation, 1 μl 1 M NAI stock solution in DMSO was added, controls received instead neat DMSO. Reactions were allowed to proceed for 10 min at the desired temperature. Then the RNA was precipitated by adding NaCl to 50 mM final concentration plus 1 μl GlycoBlue (Ambion) and ethanol, and after washing with 70% ethanol the pellet was dissolved in 10 μl TE buffer. Primer extensions were performed using ThermoScript reverse transcriptase largely as recommended by the manufacturer (ThermoFisher). In brief, 1 pmol in 3 μl of a 5´ ^32^P-labeled oligonucleotide complementary to the pUC19T7-HBVε sequence 5´-CGTCGTGACTGGGAAAAC located 35–52 nt downstream of the HBV insert (Fig 3B) was annealed to 0.1 μg of acylated RNA in 10 μl TE buffer by sequential incubation for 5 min at 65°C, 5 min at 37°C and 1 min on ice. Next, 4 μl 5x cDNA buffer (ThermoFisher), 1 μl 0.1 M DTT and 2 μl 10 mM dNTPs were added. After a 1 min preincubation at 52°C, 1 μl (15 U) ThermoScript reverse transcriptase was added and extensions were performed for 10 min at 52°C. Sequencing ladders were generated in the same way, except that the reactions were supplemented with one dideoxyNTP (ddNTP), namely 0.5 mM ddATP, ddCTP or ddTTP, or 0.125 mM ddGTP. Reactions were stopped by adding 1 μl 4 M NaOH and incubation at 90°C for 5 min. Next, 29 μl formamide loading buffer (ThermoFisher) were admixed per reaction and heating was continued for 5 min at 95°C. 10 μl aliquots of the resulting ^32^P-labeled cDNAs were resolved on 7 M urea containing 8% polyacrylamide gels run in 0.5x TBE buffer and visualized by autoradiography and/or phosphorimaging.

For on-bead SHAPE analysis approximately 0.5 μg HBV miniP-BAP bound to 15 μl (gel bed) Monomeric-Avidin-Agarose beads in 200 μl 25 mM Tris-HCl [pH 8.0], 2.5 mM DTT and 5 mM MgCl_2_ was incubated on a rotary shaker with 1 μg ε1a RNA for 3 h at 37°C. Unbound RNA was removed by three washes with NaPi/NaCl buffer (50 mM sodium phosphate [pH 6.8], 200 mM NaCl), and the beads were resuspended by adding 15 μl NaPi/NaCl buffer. Next, 0.5 μl 1 M NAI was added; the control reaction was performed in the presence of empty beads. After 5 min at 37°C RNAs were released from the beads by adding 150 μl phenol, 115 μl H_2_O and 15 μl 3 M sodium acetate [pH 5.2]. After mixing and phase separation by centrifugation RNAs in the aqueous phase were precipitated by adding 1 μl GlycoBlue and 150 μl isopropanol. The dried pellet was finally dissolved in 10 μl RNase free TE buffer, and an aliquot was subjected to primer extension as described above.

### Replication assays

Analysis of viral gene products from transfected cells by native agarose gel electrophoresis (NAGE) and subsequent immunoblotting with anti-HBc antibody mAb 312 or molecular hybridization with a ^32^P-labeled HBV DNA probe, and Southern blotting of viral particle associated DNAs using the same ^32^P-HBV probe were all performed as previously described [72]. Experimental adaptations for the in-cell SELEX procedure are described in S2 Fig.

### In vitro priming

Priming reactions were essentially performed as described for DHBV [26]. In brief, for priming with full-length HBV polymerase plasmid pT7AMVHpol_His10 was used as template in an RRL-based coupled in vitro transcription/translation system (Promega). After 60 min at 30°C the respective ε RNA was added to a final concentration of 1 μM, unless indicated otherwise, and RNP formation was allowed for another 60 min at 30°C. Actual priming was initiated by adding 1 volume of priming buffer A (20 mM Tris-HCl^-^ [pH 8.0], 20 mM NH_4_Cl, 12 mM MgCl_2_, 5 mM MnCl_2_, 0.4% NP-40 [v/v], 0.12% β-mercaptoethanol [v/v], 1 mM spermidine) plus 2 μCi of the desired α^32^P-dNTP (specific activity 3,000 Ci/mMol). For priming with bacterially expressed HBV miniP protein about 5 μg of the refolded miniP protein was mixed with in vitro transcribed or synthetic split ε RNA (annealed by slowly cooling down a heated mix of 5 μM solutions of the two RNA oligonucleotides) and supplemented with 0.5 volumes of priming buffer B (50 mM Tris-HCl [pH 8.0]; 50 mM NaCl, 0.1 mM EDTA [pH 7.5], 2.5 mM MnCl_2_) containing 2 μCi of the desired α^32^P-dNTP. After 60 min at 37°C reactions were terminated by boiling in SDS sample buffer, followed by SDS-PAGE and autoradiography.

### Data reproducibility

Reproducibility of SHAPE data was confirmed by the generation of virtually superimposable band patterns for a given RNA in three or more independent experiments (compare Figs 3, S4, S5, and S8). Data concerning replication-competence of mutant ε sequences (Figs 2, S2, S3 and S6) and principal in vitro priming capacity with different dNTPs (Figs 4, 5, 6 and 7) were semiquantitatively evaluated by visual comparison of band intensities relative to positive and negative controls run side-by-side on the same blots, all giving consistent inter-experiment rankings.

## Supporting information

S1 Fig Replication-dependent in-cell SELEX procedure for functional non-wildtype ε sequences. **(A) A library of HBV expression vectors with site-specifically randomized 5´ ε sequences.** An initial pool of ds DNAs with randomized ε upper stem positions N1-N4 and N5-N8 was generated by PCR1 using three synthetic HBV oligonucleotides of which HeRandUS+ carried the mutations; HeRand(+)Sal deliberately lacked 2 HBV nt downstream the Sal I site that are present in pCH-9/3091, yielding plasmid backbone pCH-9/3093. The PCR1 products were used as (+)-sense primer together with (-)-sense oligo HBV 20987- on plasmid pCH-9/190 which is analogous to pCH-9/3091 [52] but harbors 5´ proximal Hind III and Cla I restriction sites as markers; these will be absent in the desired PCR1 primed 1.6 kb PCR2 products. Next, the PCR2 product pool was cloned via the Sal I and Avr II (HBV position 1460) sites near the termini into plasmid pCH-9/3091_Δ3´ε_DHBVstuffPsh in which the HBV sequence between the Sal I and PshA I (pos. 494) sites was replaced by a 3.4 kb DHBV-derived stuffer fragment for easy distinction from parental plasmid. Due to a deletion in 3´ ε all ε sequences in the resulting pCH-9/3093_Δ3´ε_PCR2 pool must derive from the PCR2 pool, minimizing potential contamination with wt ε. The actually used pool was derived from ~16.000 individual bacterial colonies. **(B) In-cell SELEX procedure.** Transfection of the HBV vector pool should yield a corresponding pool of pgRNAs; only those with functional ε sequences (green) are encapsidated and can give rise to viral DNAs. To retrieve these DNAs, cytoplasmic nucleocapsids and extracellular particles were harvested. Nonencapsidated intracellular plasmid DNA from transfection was degraded by micrococcal nuclease; DNA in polyethylenglycol (PEG) precipitated nonenveloped nucleocapsids from the supernatant was, in addition to free plasmid, degraded by pronase (destroying the capsid shell) plus DNase prior to micrococcal nuclease treatment. Viral DNA from the nucleocapsids and virions was then released as for Southern blotting by SDS plus proteinase K (to degrade the covalently bound polymerase) and isolated using the QIAamp DNA Mini kit. The resulting protein-free HBV DNA was subsequently PCR amplified using primer HeRand(+)Sal with (-)-sense primer HBV20987- or, to also cover DNA derived from the major pgRNA splice product SP1, primer HBV 2833rev. On intact HBV DNA the latter primer generates a nearly 3 kb amplicon which covers additional splice sites such as SP3. Experimental data on generation of the HBV expression vector library and results of the in-cell SELEX procedure are shown in S2 Fig.
(PDF)

S2 Fig Rapid selection of wild-type ε containing HBV DNA during replication-dependent in-cell SELEX. **(A) Replication markers in Huh7 cells from the starting pool of HBV vectors with randomized ε upper stem.** Huh7 cells were transfected with wt HBV vector pCH-9/3091 or with aliquots a and b from the initial vector pool with randomized ε upper stem positions N1-N4 and N5-N8 (see Fig 1B). Four days post transfection, cytoplasmic extracts were analyzed by NAGE for capsids (by anti-HBc immunoblot) and for capsid associated viral DNA (by hybridization with a ^32^P-HBV DNA probe). Signals indicated that at least a fraction of the pool DNA encoded functional genomes. Southern blotting of DNA isolated from intracellular capsids and extracellular particles confirmed the formation of viral DNA of comparable size as seen for wt HBV, though at roughly 10-fold lower levels. Near-full-length PCR using the indicated primers produced in all cases an amplicon of the expected 2.8 kb full-length size, and in addition a major 1.6 kb product plus some weaker bands, by sequencing identified as DNAs derived from pgRNA splice products SP1 and SP3. **(B) Selection of wt-like ε sequences through four rounds of in-cell selection.** The top chromatogram shows the ε upper stem sequence of the starting pool (rd ø). The round 1 pool sequence was derived from the first round transfection PCR amplicons shown in A, with some enrichment of T at the N8 position. The amplicons were also used to produce the next generation pool of HBV vectors (see S1 Fig) which was subjected to the same procedure, and selection was repeated for two more rounds. Heterogeneity at the random positions was already low after 3 rounds, and in the round 4 pool only the wt ε sequence remained detectable. Hence mostly the round 2 and 3 pools were used as sources for individual non-wt ε sequences.
(PDF)

S3 Fig Replication competence of in-cell SELEX derived HBVs with non-wild-type ε. Individual HBV ε variant plasmids were transfected into Huh7 cells alongside wt HBV vector pCH-9/3091. The capsid immunoblot on the top and the Southern blot of intracellular capsid DNA are the same as in Fig 2B, except a larger section of the Southern blot autoradiogram is shown. The second panel from the top shows viral DNA inside capsids separated by NAGE in parallel to the immunoblot samples but detected by hybridization with a ^32^P-HBV DNA probe. The bottom panel shows the Southern blot signals for HBV DNAs isolated from extracellular particles. While a quantitative evaluation was not intended the signal intensities closest to wt were generated by variants ε0 and ε2, most apparent in the bottom panel (red arrows). As schematically shown on the right, these variants differ at only one (ε0) or two positions (ε2) from wt ε and have almost wt-ε like predicted stabilities. Variant ε1 carried three mutations, was predicted to be less stable and still performed well in the replication assays; it was therefore used in further experiments.
(PDF)

S4 Fig Enzymatic versus SHAPE secondary structure probing of ε wt and ε1 RNA. **(A) Enzymatic probing.** Nuclease accessibility provided clearly less resolution than SHAPE. Pyrimidine-specific RNase A targeted C14, U15, U18 and C19 in the bulge and U32 in the loop (*red arrows*); in ε1 RNA, the mutant residues a27 and c40 were additionally hit. More detail was provided by the nucleotide nonspecific RNase T2 (*blue arrows*) which uncovered the loop in ε wt and the more open structure of the stem below. Notably, also A13 was well recognized, in contrast to its classically being viewed as part of the top base-pair of the lower stem. However, neither residues U48-G50 opposite the bulge nor unpaired U43 and its nearest neighbors gave signals, probably because they are only accessible to a small chemical but not to a bulky enzyme. Notably, the structure-specific RNase V1 cleaved the apical part of the upper stem not only in ε wt yet also in ε1 RNA (*green Vs*), suggesting the region is structured but not canonically basepaired and/or it can adopt two or more structures with similar energy. **(B) SHAPE analysis.** The complete autoradiogram of which the 37°C and 45°C lanes are depicted in Fig 3 is shown, with the chemical mapping of accessible RNA 2´ hydroxyls to NAI. The classical bulge and loop were clearly identified (*red arrowheads*) but additional signals (*blue arrowheads*) revealed a more open bulge region and around the unpaired U43 in both ε wt and ε1 RNAs. In line with a more open upper stem ε1 RNA generated additional signals around the u27 and a41 residues. SHAPE provided much higher resolution structural information and was used throughout.
(PDF)

S5 Fig Renaturing preparation of recombinant MBP-HBV-miniP-H_6_ protein. **(A) Domain structure of the MBP-HBV-miniP-H**_**6**_ **protein.** HBV P protein residues 602–832 comprising the C terminal part of the RT domain and the RH domain were replaced by a His_6_ tag. Residue I601 in HBV P, about 60 positions downstream the YMDD active site motif, aligns with W575 in DHBV P (Lauber et al, 2017); truncation after W575 yields DHBV miniP protein with robust chaperone-independent in vitro priming activity [26]. In addition, spacer residues 200–291 were replaced by the peptide ENLYFQ; this HBV miniP protein was fused to the C terminus of N terminally His_6_-tagged maltose binding protein (MBP). **(B) Expression testing.** Plasmid pET-MBP-HP1-199_292-601H6 encoding the MBP-HBV-miniP-H_6_ ORF under control of the T7 promoter was transformed into BL21*Cp cells. After inducing expression using 0.5 mM isopropyl β-d-1-thiogalactopyranoside (IPTG), cells were shaken at 20°C overnight, harvested by centrifugation and lysed as previously described using lysozyme and benzonase in Triton X-100 containing lysis buffer plus sonication. Aliquots of the resulting suspension were directly dissolved in SDS-PAGE sample buffer (lanes “total”) or from the supernatant (SN) and pellet (P) after centrifugation at 4,000 g. BL21*Cp cells expressed massive amounts of a protein of the expected molecular mass (*arrow*), however nearly exclusively in the insoluble pellet. AE cells showed, in addition to very strong Cpn60 signals, much weaker bands at about 100 kDa, however also in the soluble SN fraction. **(C) Preparation of MBP-HBV-miniP-H**_**6**_ **inclusion bodies (IBs).** Cells from an induced 1 L BL21*Cp culture were lysed as in (B) and centrifuged immediately (yielding P0), or after 30 min on ice (yielding SN and P30). The inclusion body pellet was repeatedly triturated with wash buffer (50 mM Tris/Cl^-^ [pH 8.0], 50 mM NaCl, 5 mM DTT, 0.5 mM EDTA, 5% (v/v) glycerol, 1% (v/v) Triton X-100) and centrifuged, giving the wash fractions W1 to W3; the last wash, without Triton X-100, yielded W4 and the washed pellet Pw. Pw was finally taken up in denaturation buffer (7 M guanidium hydrochloride [GuHCl] in 50 mM Tris/Cl^-^ [pH8.0], 0.5 mM EDTA, 10 mM tris(2-carboxyethyl) phosphine [TCEP]; 3 ml for the IB pellet from a 1 L culture); the supernatant after a final centrifugation step (TLA45 rotor, 2 h at room temperature at 125.000 g) was stored in aliquots at -80°C until further use in the renaturation protocol, essentially as described for DHBV miniP [26], including reduction, e.g. with TCEP, immediately before dilution into the renaturation buffer; this likely relates to the high cysteine content of the polymerases (13 in DHBV and 19 HBV polymerase). An HBV miniP protein with all 9 of the remaining Cys residues replaced by Ala or Ser was expressed to similar levels but never yielded any priming activity; hence at least some of the cysteines are functionally relevant. Typically 2 μl of the solubilized IB preparation (containing 5–6 μg miniP) were rapidly diluted into 100 μl refolding buffer (50 mM Tris/Cl^-^ [pH 8.0], 50 mM NaCl, 1.5 M NDSB201) and kept on ice for 1 h. Then the desired ε RNA was added, usually to 0.1 mM final concentration. For in vitro priming, to 10 μl of this mixture 5 μl of priming mix (15 mM Tris/Cl^-,^[pH 8.0], 2.6 mM Mn^2+^) and 4 μCi of the respective α^32^P-dNTP (usually at 3,000 Ci/mMol) and optionally further dNTPs were added. After 1 h at 37°C reactions were stopped by boiling in SDS-containing sample buffer; radioactive labeling of P protein was monitored by SDS-PAGE and subsequent autoradiography and/or phosphorimaging.
(PDF)

S6 Fig Upper stem stability exceeding that of wt ε impairs in vitro priming and in-cell function. **(A) DHBV/HBV ε chimeras.** The central upper stem of Dε was replaced by the analogous region from the in vitro priming active HBV ε variant ε1 (D/Hε1) or wt ε (D/Hεwt). **(B) Functionality of chimeric D/Hε sequences in vitro and in cells.** In vitro transcribed Dε, D/Hε1 and D/Hεwt RNAs were subjected to in vitro priming with renatured miniDP protein and dGTP (*top*), or in the context of a full DHBV genome transfected into hepatoma cells and evaluated for formation of capsid-associated viral DNAs by Southern blotting (*bottom*). **(C) HBV ε variants with increased upper stem stabilities.** In HBV wt ε U25 was converted to C (U25C; creating a C-G instead of a U-g pair), or the unpaired U43 was deleted (ΔU43; promoting a contiguous double helix), or both mutations were combined (U25C_ΔU43); ΔG values were predicted by M-FOLD. **(D) Replication capacity of extra stable ε sequences.** The stabilizing mutations from (C) were introduced into full-length HBV expression vector pCH-9/3091 and the derivatives were transfected into Huh7 cells; a corresponding ε1a construct served as control. Cytoplasmic lysates were analyzed by NAGE and HBc-immunoblotting for capsid formation (*top*), and capsid-associated DNAs were analyzed by Southern blotting (*bottom*). Note that all vectors produced similar amounts of capsids which however contained only little (U25C) or no detectable HBV DNA (ΔU43 and U25C_ΔU43). The lower signals from the ε1a construct are in line with the rapid selection of the wt ε sequence during the in-cell SELEX, indicating that factors beyond initiation affect the overall replication performance.
(PDF)

S7 Fig Mutant ε sequences ε1 and ε1a generate ε wt-like (-)-DNA 5´ ends in cells. The ε1 and ε1a mutations were introduced into the 5´ ε sequence encoded in wt HBV expression vector pCH-9/3091. The vectors were transfected into Huh7 cells (10 cm diameter dish format). Four days post transfection viral DNA from intracellular nucleocapsids was isolated as for Southern blotting, including proteinase K digestion of the covalently bound polymerase to short peptides, in a total volume of 60 μl per transfection. **(A) Assay scheme.** Authentic (-)-strand DNA synthesis proceeds by transfer of the oligonucleotide primer generated at 5´ ε of pgRNA (*red*) to the 3´ DR1* and extension from there (*blue*). A 5´ ^32^P-labeled HBV-specific (+)-sense oligonucleotide (HBV 2945+; magenta) was then used for primer extension (*green*) on (-)-DNA, theoretically to the very 5´ terminal nt of the (-)-DNA template; however, this may be affected by remnants of the HBV polymerase, leaving an uncertainty of 1 or 2 nt. **(B) Analysis of extension products by denaturing polyacrylamide gel electrophoresis**. Extension reactions were performed in a total volume of 10 μl 1x ThermoPol buffer (NEB) containing 6 μl viral DNA, 0.2 mM dNTPs, 0.5 pmol 5´-^32^P labeled primer HBV 2945+, and 1 U Vent (exo^-^) polymerase (NEB). Primer elongation was done in a thermocycler using a temperature profile of 96°C 1 min; 45°C 5 min; 60°C 1 min; and 4°C until further use. Sequencing ladders were generated using the same primer on linearized plasmid pCH-9/3091 and dideoxy sequencing as described [19]. Regardless of the exact (-)-DNA initiation position the patterns of the extension products were very similar, most evident when comparing lane ε1 with lane wt. These data further corroborate the wt-like functionality of the in vitro priming-active ε variants ε1 and ε1a which are therefore valid models for authentic ε.
(PDF)

S8 Fig Native preparation of immobilized recombinant MBP-HBV-miniP-BAP protein. **(A) Domain structure of the MBP-HBV-miniP-BAP protein.** The protein is identical to MBP-HBV-miniP-H6 (S5 Fig) except that the C terminal His_6_ tag is replaced by a Biotin-Acceptor-Peptide (BAP; also known as Avi-tag); its central K residue can be biotinylated by *E*. *coli* BirA biotin ligase. **(B) Expression testing.** Plasmid pET-MBP-HP1-199_292-601-BAP encoding the MBP-HBV-miniP-BAP ORF under control of the phage T7 RNA polymerase promoter was transformed into BL21*Cp cells, Arctic Express (AE) cells (Agilent), or SHuffle T7 Express cells (NEB); note that AE cells express large amounts of the cold shock chaperonins Cpn60 (*arrow*) and Cpn10. After induction using 0.5 mM IPTG BL21*Cp and SHuffle Express cells were shaken at 20°C and AE cells at 12°C overnight. Small aliquots of cells without vs. with IPTG were directly lysed in SDS-PAGE sample buffer (SDS lysate -/+); the bulk of cells was lysed as described in S5 Fig and aliquots of the crude lysate (total) and the supernatant (SN) and pellet (P) after centrifugation were analzyed by SDS-PAGE and subsequent immunoblotting with peroxidase (PO) conjugated streptavidin, followed by enhanced chemiluminescent (ECL) substrate. Only the SN sample from AE sample contained a reactive ~100 kDa band. **(C) Enrichment of immobilized MBP-HBV-miniP-BAP protein.** Cleared lysate from the induced AE culture was passed through a small column (5 ml volume for lysate from a 300 ml culture) filled with Pierce monomeric avidin agarose (Thermo Scientific). Column preparation, sample loading and washing (lane W) were performed as recommended by the manufacturer. A fraction of the 100 kDa protein could be eluted (lane E), together with excess Cpn60, by 2 mM D-biotin in wash buffer (100 mM Na^+^ phosphate, 150 mM NaCl, pH 7.0). More of the 100 kDa protein plus Cpn60 and various less abundant proteins remained on the beads, as shown by their release via boiling in SDS-PAGE sample buffer (lane B). In subsequent experiments the D-biotin elution step was omitted. Instead the washed beads were stored, after adding EDTA-free protease inhibitor cocktail (Roche), in small aliquots at -80°C. For on-bead in vitro priming, about 5 μl beads (~200 ng miniP) were adjusted in a total volume of 10 μl to 25 mM Tris/Cl^-^ [pH 8.0], 2.5 mM DTT, 5 mM MgCl_2_, 1 U/μl RNasin (Promega) and typically 1 μM ε RNA. After 1 h at 37°C 5 μl priming mix were added as described in Fig 5 for renatured HBV-miniP protein.
(PDF)
