# Supplementary material for: Relaxing the restricted structural dynamics in the human hepatitis B virus RNA encapsidation signal enables replication initiation in vitro
Source: PLoS Pathog. 2022 Mar 8;18(3):e1010362. doi: 10.1371/journal.ppat.1010362 (PMC8903280; doi:10.1371/journal.ppat.1010362)
Supplement: S1 Fig — (A) A library of HBV expression vectors with site-specifically randomized 5´ ε sequences. An initial pool of ds DNAs with randomized ε upper stem positions N1-N4 and N5-N8 was generated by PCR1 using three synthetic HBV oligonucleotides of which HeRandUS+ carried the mutations; HeRand(+)Sal deliberately lacked 2 HBV nt downstream the Sal I site that are present in pCH-9/3091, yielding plasmid backbone pCH-9/3093. The PCR1 products were used as (+)-sense primer together with (-)-sense oligo HBV 20987- on plasmid pCH-9/190 which is analogous to pCH-9/3091 [52] but harbors 5´ proximal Hind III and Cla I restriction sites as markers; these will be absent in the desired PCR1 primed 1.6 kb PCR2 products. Next, the PCR2 product pool was cloned via the Sal I and Avr II (HBV position 1460) sites near the termini into plasmid pCH-9/3091_Δ3´ε_DHBVstuffPsh in which the HBV sequence between the Sal I and PshA I (pos. 494) sites was replaced by a 3.4 kb DHBV-derived stuffer fragment for easy distinction from parental plasmid. Due to a deletion in 3´ ε all ε sequences in the resulting pCH-9/3093_Δ3´ε_PCR2 pool must derive from the PCR2 pool, minimizing potential contamination with wt ε. The actually used pool was derived from ~16.000 individual bacterial colonies. (B) In-cell SELEX procedure. Transfection of the HBV vector pool should yield a corresponding pool of pgRNAs; only those with functional ε sequences (green) are encapsidated and can give rise to viral DNAs. To retrieve these DNAs, cytoplasmic nucleocapsids and extracellular particles were harvested. Nonencapsidated intracellular plasmid DNA from transfection was degraded by micrococcal nuclease; DNA in polyethylenglycol (PEG) precipitated nonenveloped nucleocapsids from the supernatant was, in addition to free plasmid, degraded by pronase (destroying the capsid shell) plus DNase prior to micrococcal nuclease treatment. Viral DNA from the nucleocapsids and virions was then released as for Southern blotting by SDS plus pro [file ppat.1010362.s001.pdf]

S1 Fig

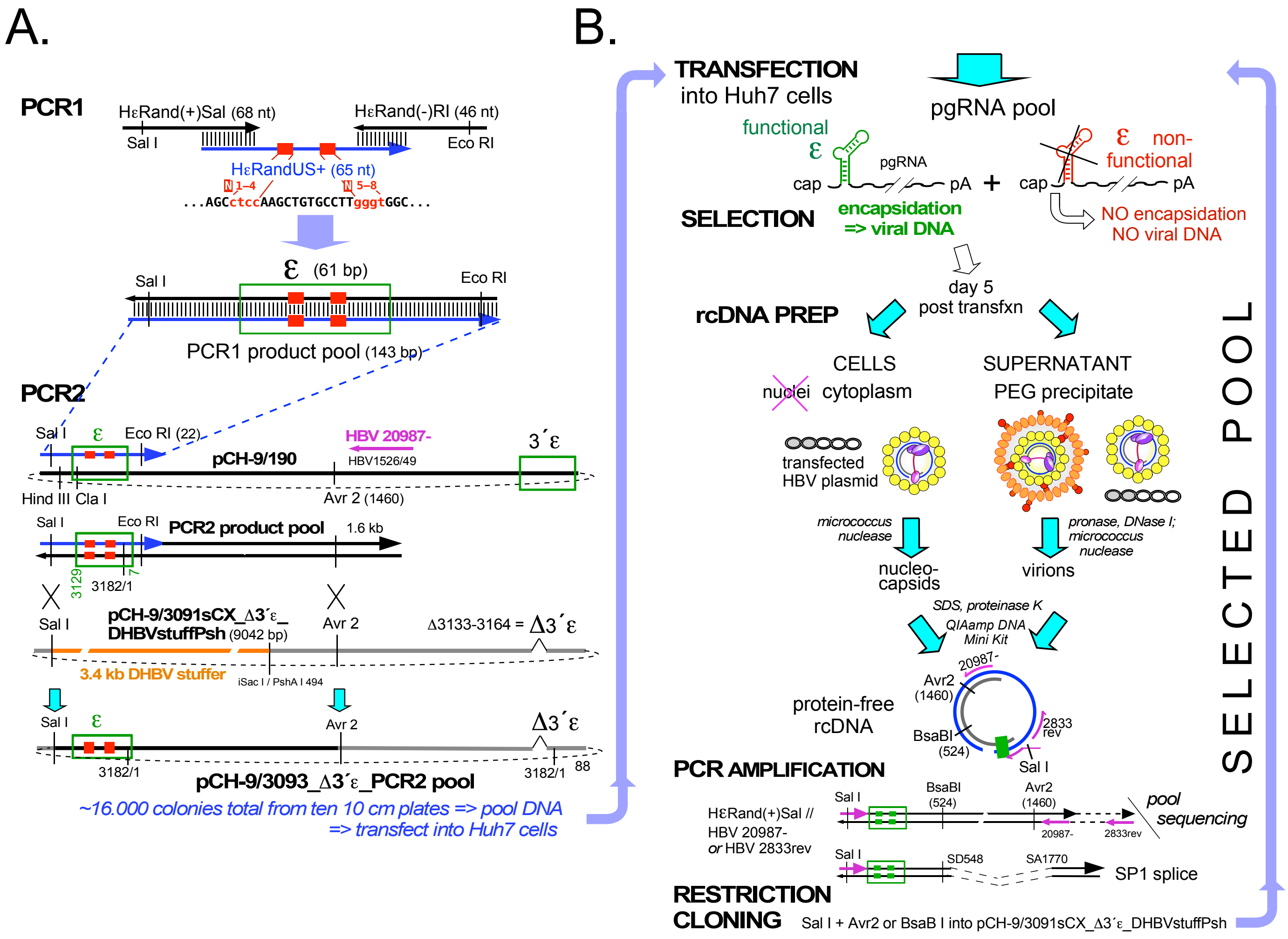

S1 Fig. Replication-dependent in-cell SELEX procedure for functional non-wildtype ε sequences. (A) A library of HBV expression vectors with site-specifically randomized 5´ ε sequences. An initial pool of ds DNAs with randomized ε upper stem positions N1-N4 and N5-N8 was generated by PCR1 using three synthetic HBV oligonucleotides of which HeRandUS+ carried the mutations; HeRand(+)Sal deliberately lacked 2 HBV nt downstream the Sal I site that are present in pCH-9/3091, yielding plasmid backbone pCH-9/3093. The PCR1 products were used as (+)-sense primer together with (-)-sense oligo HBV 20987- on plasmid pCH-9/190 which is analogous to pCH-9/3091 [52] but harbors 5´ proximal Hind III and Cla I restriction sites as markers; these will be absent in the desired PCR1 primed 1.6 kb PCR2 products. Next, the PCR2 product pool was cloned via the Sal I and Avr II (HBV position 1460) sites near the termini into plasmid pCH-9/3091_Δ3´ε_DHBVstuffPsh in which the HBV sequence between the Sal I and PshA I (pos. 494) sites was replaced by a 3.4 kb DHBV-derived stuffer fragment for easy distinction from parental plasmid. Due to a deletion in 3´ ε all ε sequences in the resulting pCH-9/3093_Δ3´ε_PCR2 pool must derive from the PCR2 pool, minimizing potential contamination with wt ε. The actually used pool was derived from ~16.000 individual bacterial colonies. **(B) In-cell SELEX procedure.** Transfection of the HBV vector pool should yield a corresponding pool of pgRNAs; only those with functional ε sequences (green) are encapsidated and can give rise to viral DNAs. To retrieve these DNAs, cytoplasmic nucleocapsids and extracellular particles were harvested. Nonencapsidated intracellular plasmid DNA from transfection was degraded by micrcoccal nuclease; DNA in polyethylenglycol (PEG) precipitated nonenveloped nucleocapsids from the supernatant was, in addition to free plasmid, degraded by pronase (destroying the capsid shell) plus DNase prior to micrococcal nuclease treatment. Viral DNA from the nucleocapsids and virions was then released as for Southern blotting by SDS plus proteinase K (to degrade the covalently bound polymerase) and isolated using the QIAamp DNA Mini kit. The resulting protein-free HBV DNA was subsequently PCR amplified using primer HeRand(+)Sal with (-)-sense primer HBV20987- or, to also cover DNA derived from the major pgRNA splice product SP1, primer HBV 2833rev. On intact HBV DNA the latter primer generates a nearly 3 kb amplicon which covers additional splice sites such as SP3. Experimental data on generation of the HBV expression vector library and results of the in-cell SELEX procedure are shown in S2 Fig.
