# Supplementary material for: Relaxing the restricted structural dynamics in the human hepatitis B virus RNA encapsidation signal enables replication initiation in vitro
Source: PLoS Pathog. 2022 Mar 8;18(3):e1010362. doi: 10.1371/journal.ppat.1010362 (PMC8903280; doi:10.1371/journal.ppat.1010362)
Supplement: S2 Fig — (A) Replication markers in Huh7 cells from the starting pool of HBV vectors with randomized ε upper stem. Huh7 cells were transfected with wt HBV vector pCH-9/3091 or with aliquots a and b from the initial vector pool with randomized ε upper stem positions N1-N4 and N5-N8 (see Fig 1B). Four days post transfection, cytoplasmic extracts were analyzed by NAGE for capsids (by anti-HBc immunoblot) and for capsid associated viral DNA (by hybridization with a 32P-HBV DNA probe). Signals indicated that at least a fraction of the pool DNA encoded functional genomes. Southern blotting of DNA isolated from intracellular capsids and extracellular particles confirmed the formation of viral DNA of comparable size as seen for wt HBV, though at roughly 10-fold lower levels. Near-full-length PCR using the indicated primers produced in all cases an amplicon of the expected 2.8 kb full-length size, and in addition a major 1.6 kb product plus some weaker bands, by sequencing identified as DNAs derived from pgRNA splice products SP1 and SP3. (B) Selection of wt-like ε sequences through four rounds of in-cell selection. The top chromatogram shows the ε upper stem sequence of the starting pool (rd ø). The round 1 pool sequence was derived from the first round transfection PCR amplicons shown in A, with some enrichment of T at the N8 position. The amplicons were also used to produce the next generation pool of HBV vectors (see S1 Fig) which was subjected to the same procedure, and selection was repeated for two more rounds. Heterogeneity at the random positions was already low after 3 rounds, and in the round 4 pool only the wt ε sequence remained detectable. Hence mostly the round 2 and 3 pools were used as sources for individual non-wt ε sequences. (PDF) [file ppat.1010362.s002.pdf]

S2 Fig

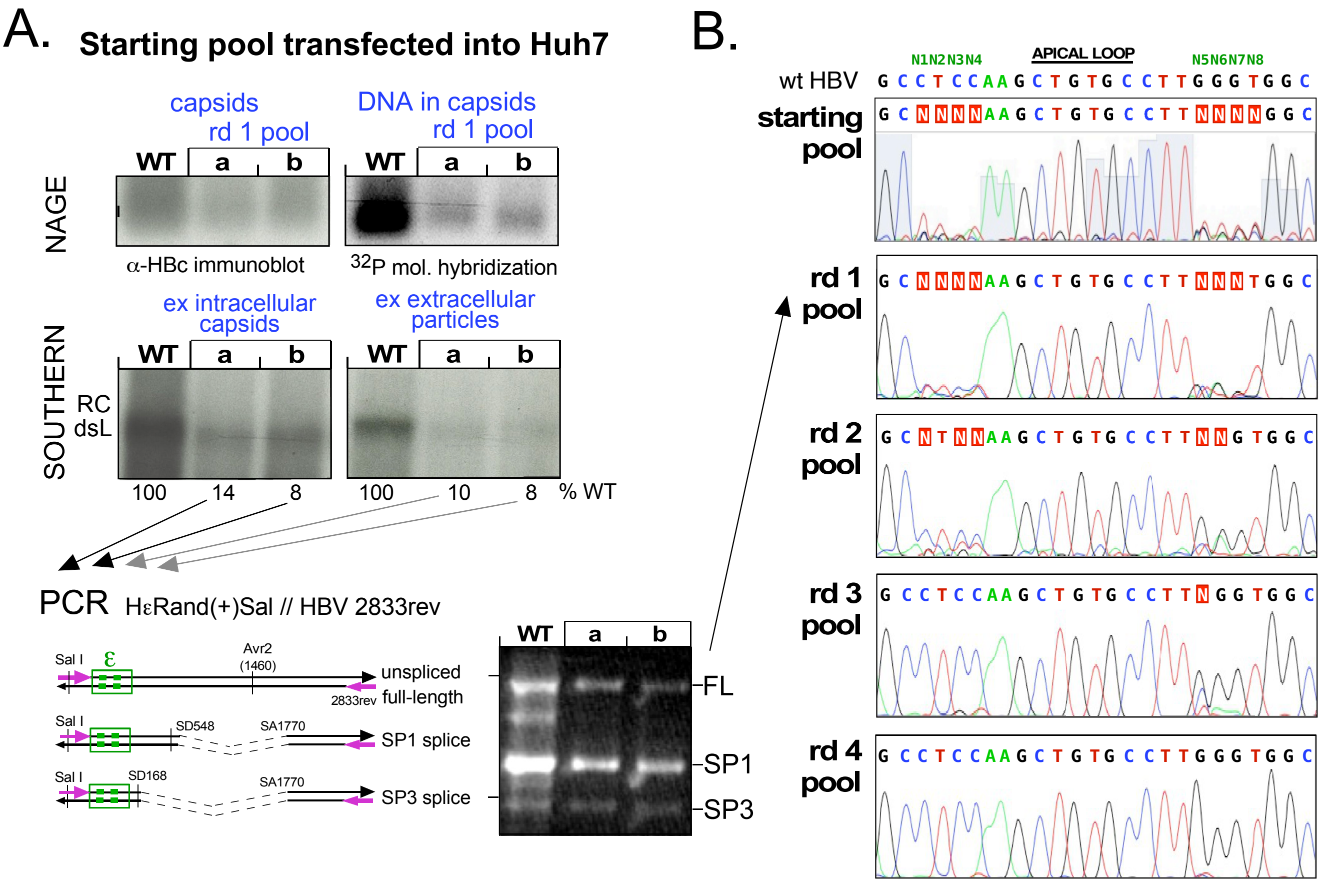

S2 Fig. Rapid selection of wild-type ε containing HBV DNA during replication-dependent in-cell SELEX. (A) Replication markers in Huh7 cells from the starting pool of HBV vectors with randomized ε upper stem. Huh7 cells were transfected with wt HBV vector pCH-9/3091 or with aliquots a and b from the initial vector pool with randomized ε upper stem positions N1-N4 and N5-N8 (see Fig 1B). Four days post transfection, cytoplasmic extracts were analyzed by NAGE for capsids (by anti-HBc immunoblot) and for capsid associated viral DNA (by hybridization with a ^{32}P-HBV DNA probe). Signals indicated that at least a fraction of the pool DNA encoded functional genomes. Southern blotting of DNA isolated from intracellular capsids and extracellular particles confirmed the formation of viral DNA of comparable size as seen for wt HBV, though at roughly 10-fold lower levels. Near-full-length PCR using the indicated primers produced in all cases an amplicon of the expected 2.8 kb full-length size, and in addition a major 1.6 kb product plus some weaker bands, by sequencing identified as DNAs derived from pgRNA splice products SP1 and SP3. **(B) Selection of wt-like ε sequences through four rounds of in-cell selection.** The top chromatogram shows the ε upper stem sequence of the starting pool (rd ø). The round 1 pool sequence was derived from the first round transfection PCR amplicons shown in A, with some enrichment of T at the N8 position. The amplicons were also used to produce the next generation pool of HBV vectors (see S1 Fig) which was subjected to the same procedure, and selection was repeated for two more rounds. Heterogeneity at the random positions was already low after 3 rounds, and in the round 4 pool only the wt ε sequence remained detectable. Hence mostly the round 2 and 3 pools were used as sources for individual non-wt ε sequences.
