# Supplementary material for: Relaxing the restricted structural dynamics in the human hepatitis B virus RNA encapsidation signal enables replication initiation in vitro
Source: PLoS Pathog. 2022 Mar 8;18(3):e1010362. doi: 10.1371/journal.ppat.1010362 (PMC8903280; doi:10.1371/journal.ppat.1010362)
Supplement: S3 Fig — Individual HBV ε variant plasmids were transfected into Huh7 cells alongside wt HBV vector pCH-9/3091. The capsid immunoblot on the top and the Southern blot of intracellular capsid DNA are the same as in Fig 2B, except a larger section of the Southern blot autoradiogram is shown. The second panel from the top shows viral DNA inside capsids separated by NAGE in parallel to the immunoblot samples but detected by hybridization with a 32P-HBV DNA probe. The bottom panel shows the Southern blot signals for HBV DNAs isolated from extracellular particles. While a quantitative evaluation was not intended the signal intensities closest to wt were generated by variants ε0 and ε2, most apparent in the bottom panel (red arrows). As schematically shown on the right, these variants differ at only one (ε0) or two positions (ε2) from wt ε and have almost wt-ε like predicted stabilities. Variant ε1 carried three mutations, was predicted to be less stable and still performed well in the replication assays; it was therefore used in further experiments. (PDF) [file ppat.1010362.s003.pdf]

S3 Fig

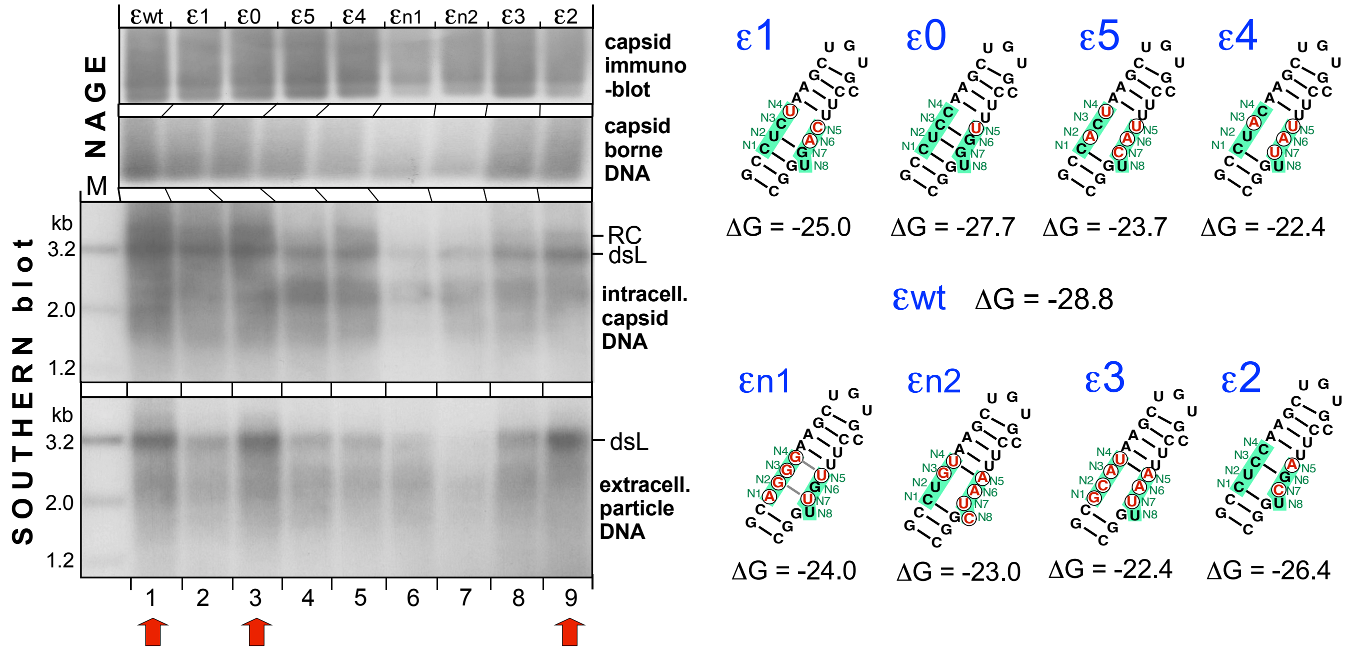

S3 Fig. Replication competence of in-cell SELEX derived HBVs with non-wild-type ε. Individual HBV ε variant plasmids were transfected into Huh7 cells alongside wt HBV vector pCH-9/3091. The capsid immunoblot on the top and the Southern blot of intracellular capsid DNA are the same as in Fig 2B, except a larger section of the Southern blot autoradiogram is shown. The second panel from the top shows viral DNA inside capsids separated by NAGE in parallel to the immunoblot samples but detected by hybridization with a ^{32}P-HBV DNA probe. The bottom panel shows the Southern blot signals for HBV DNAs isolated from extracellular particles. While a quantitative evaluation was not intended the signal intensities closest to wt were generated by variants ε0 and ε2, most apparent in the bottom panel (*red arrows*). As schematically shown on the right, these variants differ at only one (ε0) or two positions (ε2) from wt ε and have almost wt-ε like predicted stabilities. Variant ε1 carried three mutations, was predicted to be less stable and still performed well in the replication assays; it was therefore used in further experiments.
