# Supplementary material for: Relaxing the restricted structural dynamics in the human hepatitis B virus RNA encapsidation signal enables replication initiation in vitro
Source: PLoS Pathog. 2022 Mar 8;18(3):e1010362. doi: 10.1371/journal.ppat.1010362 (PMC8903280; doi:10.1371/journal.ppat.1010362)
Supplement: S4 Fig — (A) Enzymatic probing. Nuclease accessibility provided clearly less resolution than SHAPE. Pyrimidine-specific RNase A targeted C14, U15, U18 and C19 in the bulge and U32 in the loop (red arrows); in ε1 RNA, the mutant residues a27 and c40 were additionally hit. More detail was provided by the nucleotide nonspecific RNase T2 (blue arrows) which uncovered the loop in ε wt and the more open structure of the stem below. Notably, also A13 was well recognized, in contrast to its classically being viewed as part of the top base-pair of the lower stem. However, neither residues U48-G50 opposite the bulge nor unpaired U43 and its nearest neighbors gave signals, probably because they are only accessible to a small chemical but not to a bulky enzyme. Notably, the structure-specific RNase V1 cleaved the apical part of the upper stem not only in ε wt yet also in ε1 RNA (green Vs), suggesting the region is structured but not canonically basepaired and/or it can adopt two or more structures with similar energy. (B) SHAPE analysis. The complete autoradiogram of which the 37°C and 45°C lanes are depicted in Fig 3 is shown, with the chemical mapping of accessible RNA 2´ hydroxyls to NAI. The classical bulge and loop were clearly identified (red arrowheads) but additional signals (blue arrowheads) revealed a more open bulge region and around the unpaired U43 in both ε wt and ε1 RNAs. In line with a more open upper stem ε1 RNA generated additional signals around the u27 and a41 residues. SHAPE provided much higher resolution structural information and was used throughout. (PDF) [file ppat.1010362.s004.pdf]

S4 Fig

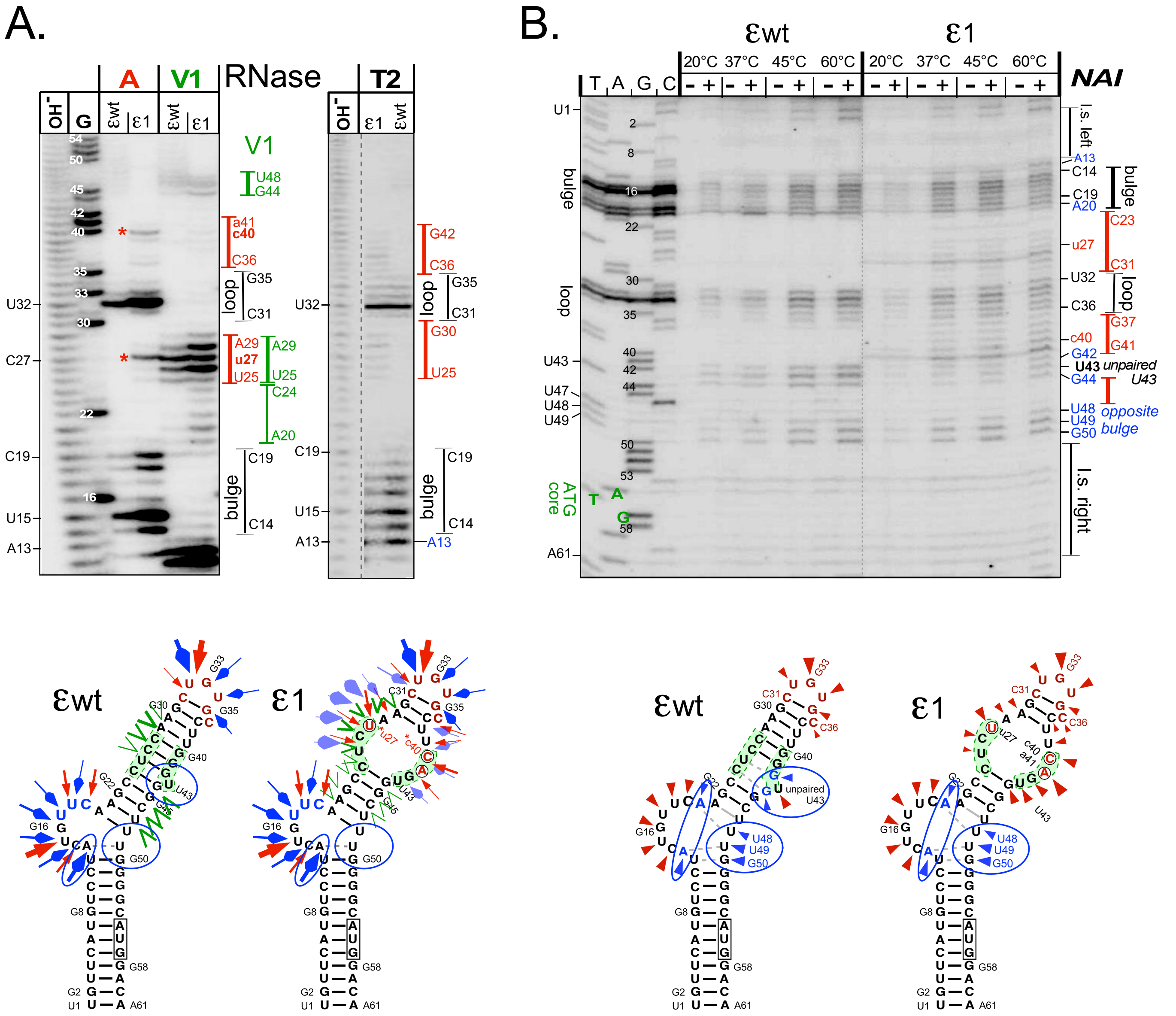

S4 Fig. Enzymatic versus SHAPE secondary structure probing of ε wt and ε1 RNA. (A) Enzymatic probing. Nuclease accessibility provided clearly less resolution than SHAPE. Pyrimidine-specific RNase A targeted C14, U15, U18 and C19 in the bulge and U32 in the loop (*red arrows*); in ε1 RNA, the mutant residues a27 and c40 were additionally hit. More detail was provided by the nucleotide nonspecific RNase T2 (*blue arrows*) which uncovered the loop in ε wt and the more open structure of the stem below. Notably, also A13 was well recognized, in contrast to its classically being viewed as part of the top base-pair of the lower stem. However, neither residues U48-G50 opposite the bulge nor unpaired U43 and its nearest neighbors gave signals, probably because they are only accessible to a small chemical but not to a bulky enzyme. Notably, the structure-specific RNase V1 cleaved the apical part of the upper stem not only in ε wt yet also in ε1 RNA (*green Vs*), suggesting the region is structured but not canonically basepaired and/or it can adopt two or more structures with similar energy. **(B) SHAPE analysis.** The complete autoradiogram of which the 37°C and 45°C lanes are depicted in Fig 3 is shown, with the chemical mapping of accessible RNA 2´ hydroxyls to NAI. The classical bulge and loop were clearly identified (*red arrowheads*) but additional signals (*blue arrowheads*) revealed a more open bulge region and around the unpaired U43 in both ε wt and ε1 RNAs. In line with a more open upper stem ε1 RNA generated additional signals around the u27 and a41 residues. SHAPE provided much higher resolution structural information and was used throughout.
