# Supplementary material for: Relaxing the restricted structural dynamics in the human hepatitis B virus RNA encapsidation signal enables replication initiation in vitro
Source: PLoS Pathog. 2022 Mar 8;18(3):e1010362. doi: 10.1371/journal.ppat.1010362 (PMC8903280; doi:10.1371/journal.ppat.1010362)
Supplement: S5 Fig — (A) Domain structure of the MBP-HBV-miniP-H6 protein. HBV P protein residues 602–832 comprising the C terminal part of the RT domain and the RH domain were replaced by a His6 tag. Residue I601 in HBV P, about 60 positions downstream the YMDD active site motif, aligns with W575 in DHBV P (Lauber et al, 2017); truncation after W575 yields DHBV miniP protein with robust chaperone-independent in vitro priming activity [26]. In addition, spacer residues 200–291 were replaced by the peptide ENLYFQ; this HBV miniP protein was fused to the C terminus of N terminally His6-tagged maltose binding protein (MBP). (B) Expression testing. Plasmid pET-MBP-HP1-199_292-601H6 encoding the MBP-HBV-miniP-H6 ORF under control of the T7 promoter was transformed into BL21*Cp cells. After inducing expression using 0.5 mM isopropyl β-d-1-thiogalactopyranoside (IPTG), cells were shaken at 20°C overnight, harvested by centrifugation and lysed as previously described using lysozyme and benzonase in Triton X-100 containing lysis buffer plus sonication. Aliquots of the resulting suspension were directly dissolved in SDS-PAGE sample buffer (lanes “total”) or from the supernatant (SN) and pellet (P) after centrifugation at 4,000 g. BL21*Cp cells expressed massive amounts of a protein of the expected molecular mass (arrow), however nearly exclusively in the insoluble pellet. AE cells showed, in addition to very strong Cpn60 signals, much weaker bands at about 100 kDa, however also in the soluble SN fraction. (C) Preparation of MBP-HBV-miniP-H6 inclusion bodies (IBs). Cells from an induced 1 L BL21*Cp culture were lysed as in (B) and centrifuged immediately (yielding P0), or after 30 min on ice (yielding SN and P30). The inclusion body pellet was repeatedly triturated with wash buffer (50 mM Tris/Cl- [pH 8.0], 50 mM NaCl, 5 mM DTT, 0.5 mM EDTA, 5% (v/v) glycerol, 1% (v/v) Triton X-100) and centrifuged, giving the wash fractions W1 to W3; the last wash, without Triton X-100, yielded W4 and the washed [file ppat.1010362.s005.pdf]

S5 Fig

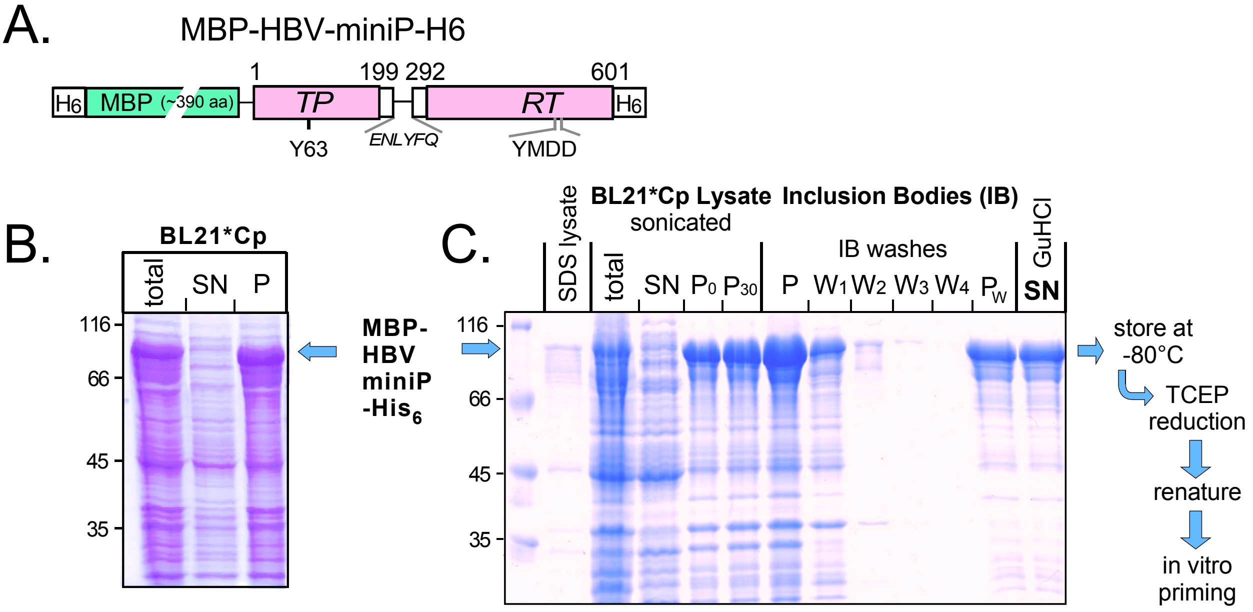

S5 Fig. Renaturing preparation of recombinant MBP-HBV-miniP-H$_6$ protein. (A) Domain structure of the MBP-HBV-miniP-H$_6$ protein. HBV P protein residues 602-832 comprising the C terminal part of the RT domain and the RH domain were replaced by a His$_6$ tag. Residue I601 in HBV P, about 60 positions downstream the YMDD active site motif, aligns with W575 in DHBV P [59]; truncation after W575 yields DHBV miniP protein with robust chaperone-independent in vitro priming activity [26]. In addition, spacer residues 200-291 were replaced by the peptide ENLYFQ; this HBV miniP protein was fused to the C terminus of N terminally His$_6$-tagged maltose binding protein (MBP). **(B) Expression testing.** Plasmid pET-MBP-HP1-199_292-601H6 encoding the MBP-HBV-miniP-H6 ORF under control of the T7 promoter was transformed into BL21*Cp cells. After inducing expression using 0.5 mM isopropyl β-d-1-thiogalactopyranoside (IPTG), cells were shaken at 20°C overnight, harvested by centrifugation and lysed as previously described using lysozyme and benzonase in Triton X-100 containing lysis buffer plus sonication. Aliquots of the resulting suspension were directly dissolved in SDS-PAGE sample buffer (lanes “total”) or from the supernatant (SN) and pellet (P) after centrifugation at 4,000 g. BL21*Cp cells expressed massive amounts of a protein of the expected molecular mass (*arrow*), however nearly exclusively in the insoluble pellet. AE cells showed, in addition to very strong Cpn60 signals, much weaker bands at about 100 kDa, however also in the soluble SN fraction. **(C) Preparation of MBP-HBV-miniP-H$_6$ inclusion bodies (IBs).** Cells from an induced 1 L BL21*Cp culture were lysed as in (B) and centrifuged immediately (yielding P0), or after 30 min on ice (yielding SN and P30). The inclusion body pellet was repeatedly triturated with wash buffer (50 mM Tris/Cl$^-$ [pH 8.0], 50 mM NaCl, 5 mM DTT, 0.5 mM EDTA, 5% (v/v) glycerol, 1% (v/v) Triton X-100) and centrifuged, giving the wash fractions W1 to W3; the last wash, without Triton X-100, yielded W4 and the washed pellet Pw. Pw was finally taken up in denaturation buffer (7 M guanidium hydrochloride [GuHCl] in 50 mM Tris/Cl$^-$ [pH8.0], 0.5 mM EDTA, 10 mM tris(2-carboxyethyl) phosphine [TCEP]; 3 ml for the IB pellet from a 1 L culture); the supernatant after a final centrifugation step (TLA45 rotor, 2 h at room temperature at 125.000 g) was stored in aliquots at -80°C until further use in the renaturation protocol, essentially as described for DHBV miniP [26], including reduction, e.g. with TCEP, immediately before dilution into the renaturation buffer; this likely relates to the high cysteine content of the polymerases (13 in DHBV and 19 HBV polymerase). An HBV miniP protein with all 9 of the remaining Cys residues replaced by Ala or Ser was expressed to similar levels but never yielded any priming activity; hence at least some of the cysteines are functionally relevant. Typically 2 μl of the solubilized IB preparation (containing 5-6 μg miniP) were rapidly diluted into 100 μl refolding buffer (50 mM Tris/Cl$^-$ [pH 8.0], 50 mM NaCl, 1.5 M NDSB201) and kept on ice for 1 h. Then the desired ε RNA was added, usually to 0.1 mM final concentration. For in vitro priming, to 10 μl of this mixture 5 μl of priming mix (15 mM Tris/Cl$^-$ [pH 8.0], 2.6 mM Mn^{2+}) and 4 μCi of the respective α^{32}P-dNTP (usually at 3,000 Ci/mMol) and optionally further dNTPs were added. After 1 h at 37°C reactions were stopped by boiling in SDS-containing sample buffer; radioactive labeling of P protein was monitored by SDS-PAGE and subsequent autoradiography and/or phosphorimaging.
