# Supplementary material for: Relaxing the restricted structural dynamics in the human hepatitis B virus RNA encapsidation signal enables replication initiation in vitro
Source: PLoS Pathog. 2022 Mar 8;18(3):e1010362. doi: 10.1371/journal.ppat.1010362 (PMC8903280; doi:10.1371/journal.ppat.1010362)
Supplement: S6 Fig — (A) DHBV/HBV ε chimeras. The central upper stem of Dε was replaced by the analogous region from the in vitro priming active HBV ε variant ε1 (D/Hε1) or wt ε (D/Hεwt). (B) Functionality of chimeric D/Hε sequences in vitro and in cells. In vitro transcribed Dε, D/Hε1 and D/Hεwt RNAs were subjected to in vitro priming with renatured miniDP protein and dGTP (top), or in the context of a full DHBV genome transfected into hepatoma cells and evaluated for formation of capsid-associated viral DNAs by Southern blotting (bottom). (C) HBV ε variants with increased upper stem stabilities. In HBV wt ε U25 was converted to C (U25C; creating a C-G instead of a U-g pair), or the unpaired U43 was deleted (ΔU43; promoting a contiguous double helix), or both mutations were combined (U25C_ΔU43); ΔG values were predicted by M-FOLD. (D) Replication capacity of extra stable ε sequences. The stabilizing mutations from (C) were introduced into full-length HBV expression vector pCH-9/3091 and the derivatives were transfected into Huh7 cells; a corresponding ε1a construct served as control. Cytoplasmic lysates were analyzed by NAGE and HBc-immunoblotting for capsid formation (top), and capsid-associated DNAs were analyzed by Southern blotting (bottom). Note that all vectors produced similar amounts of capsids which however contained only little (U25C) or no detectable HBV DNA (ΔU43 and U25C_ΔU43). The lower signals from the ε1a construct are in line with the rapid selection of the wt ε sequence during the in-cell SELEX, indicating that factors beyond initiation affect the overall replication performance. (PDF) [file ppat.1010362.s006.pdf]

S6 Fig

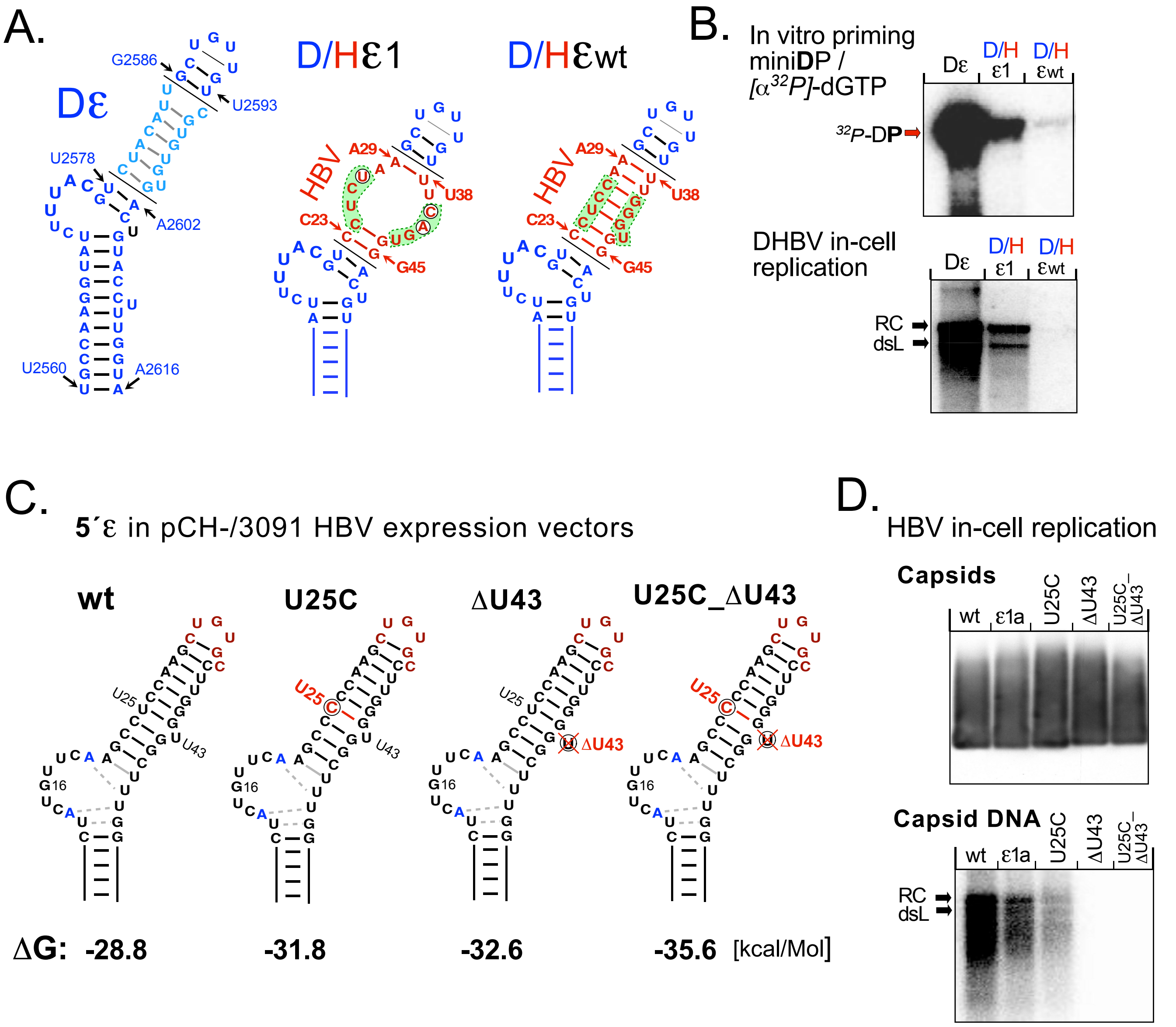

S6 Fig. Upper stem stability exceeding that of wt ε impairs in vitro priming and in-cell function. (A) DHBV/HBV ε chimeras. The central upper stem of Dε was replaced by the analogous region from the in vitro priming active HBV ε variant ε1 (D/Hε1) or wt ε (D/Hεwt). **(B) Functionality of chimeric D/Hε sequences in vitro and in cells.** In vitro transcribed Dε, D/Hε1 and D/Hεwt RNAs were subjected to in vitro priming with renatured miniDP protein and dGTP (*top*), or in the context of a full DHBV genome transfected into hepatoma cells and evaluated for formation of capsid-associated viral DNAs by Southern blotting (*bottom*). **(C) HBV ε variants with increased upper stem stabilities.** In HBV wt ε U25 was converted to C (U25C; creating a C-G instead of a U-g pair), or the unpaired U43 was deleted (ΔU43; promoting a contiguous double helix), or both mutations were combined (U25C_ΔU43); ΔG values were predicted by M-FOLD. **(D) Replication capacity of extra stable ε sequences.** The stabilizing mutations from (C) were introduced into full-length HBV expression vector pCH-9/3091 and the derivatives were transfected into Huh7 cells; a corresponding ε1a construct served as control. Cytoplasmic lysates were analyzed by NAGE and HBc-immunoblotting for capsid formation (*top*), and capsid-associated DNAs were analyzed by Southern blotting (*bottom*). Note that all vectors produced similar amounts of capsids which however contained only little (U25C) or no detectable HBV DNA (ΔU43 and U25C_ΔU43). The lower signals from the ε1a construct are in line with the rapid selection of the wt ε sequence during the in-cell SELEX, indicating that factors beyond initiation affect the overall replication performance.
