# Supplementary figures and images for: Relaxing the restricted structural dynamics in the human hepatitis B virus RNA encapsidation signal enables replication initiation in vitro
Source: PLoS Pathog. 2022 Mar 8;18(3):e1010362. doi: 10.1371/journal.ppat.1010362 (PMC8903280; doi:10.1371/journal.ppat.1010362)

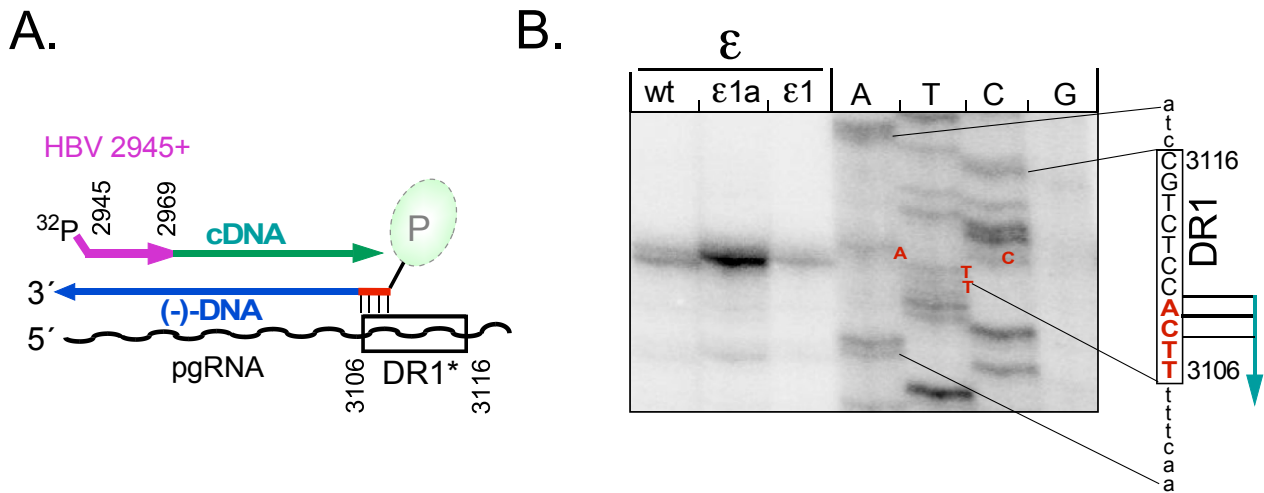

Supplement: S7 Fig — The ε1 and ε1a mutations were introduced into the 5´ ε sequence encoded in wt HBV expression vector pCH-9/3091. The vectors were transfected into Huh7 cells (10 cm diameter dish format). Four days post transfection viral DNA from intracellular nucleocapsids was isolated as for Southern blotting, including proteinase K digestion of the covalently bound polymerase to short peptides, in a total volume of 60 μl per transfection. (A) Assay scheme. Authentic (-)-strand DNA synthesis proceeds by transfer of the oligonucleotide primer generated at 5´ ε of pgRNA (red) to the 3´ DR1* and extension from there (blue). A 5´ 32P-labeled HBV-specific (+)-sense oligonucleotide (HBV 2945+; magenta) was then used for primer extension (green) on (-)-DNA, theoretically to the very 5´ terminal nt of the (-)-DNA template; however, this may be affected by remnants of the HBV polymerase, leaving an uncertainty of 1 or 2 nt. (B) Analysis of extension products by denaturing polyacrylamide gel electrophoresis. Extension reactions were performed in a total volume of 10 μl 1x ThermoPol buffer (NEB) containing 6 μl viral DNA, 0.2 mM dNTPs, 0.5 pmol 5´-32P labeled primer HBV 2945+, and 1 U Vent (exo-) polymerase (NEB). Primer elongation was done in a thermocycler using a temperature profile of 96°C 1 min; 45°C 5 min; 60°C 1 min; and 4°C until further use. Sequencing ladders were generated using the same primer on linearized plasmid pCH-9/3091 and dideoxy sequencing as described [19]. Regardless of the exact (-)-DNA initiation position the patterns of the extension products were very similar, most evident when comparing lane ε1 with lane wt. These data further corroborate the wt-like functionality of the in vitro priming-active ε variants ε1 and ε1a which are therefore valid models for authentic ε. (PDF) [file ppat.1010362.s007.pdf]
