# Supplementary material for: Relaxing the restricted structural dynamics in the human hepatitis B virus RNA encapsidation signal enables replication initiation in vitro
Source: PLoS Pathog. 2022 Mar 8;18(3):e1010362. doi: 10.1371/journal.ppat.1010362 (PMC8903280; doi:10.1371/journal.ppat.1010362)
Supplement: S8 Fig — (A) Domain structure of the MBP-HBV-miniP-BAP protein. The protein is identical to MBP-HBV-miniP-H6 (S5 Fig) except that the C terminal His6 tag is replaced by a Biotin-Acceptor-Peptide (BAP; also known as Avi-tag); its central K residue can be biotinylated by E. coli BirA biotin ligase. (B) Expression testing. Plasmid pET-MBP-HP1-199_292-601-BAP encoding the MBP-HBV-miniP-BAP ORF under control of the phage T7 RNA polymerase promoter was transformed into BL21*Cp cells, Arctic Express (AE) cells (Agilent), or SHuffle T7 Express cells (NEB); note that AE cells express large amounts of the cold shock chaperonins Cpn60 (arrow) and Cpn10. After induction using 0.5 mM IPTG BL21*Cp and SHuffle Express cells were shaken at 20°C and AE cells at 12°C overnight. Small aliquots of cells without vs. with IPTG were directly lysed in SDS-PAGE sample buffer (SDS lysate -/+); the bulk of cells was lysed as described in S5 Fig and aliquots of the crude lysate (total) and the supernatant (SN) and pellet (P) after centrifugation were analzyed by SDS-PAGE and subsequent immunoblotting with peroxidase (PO) conjugated streptavidin, followed by enhanced chemiluminescent (ECL) substrate. Only the SN sample from AE sample contained a reactive ~100 kDa band. (C) Enrichment of immobilized MBP-HBV-miniP-BAP protein. Cleared lysate from the induced AE culture was passed through a small column (5 ml volume for lysate from a 300 ml culture) filled with Pierce monomeric avidin agarose (Thermo Scientific). Column preparation, sample loading and washing (lane W) were performed as recommended by the manufacturer. A fraction of the 100 kDa protein could be eluted (lane E), together with excess Cpn60, by 2 mM D-biotin in wash buffer (100 mM Na+ phosphate, 150 mM NaCl, pH 7.0). More of the 100 kDa protein plus Cpn60 and various less abundant proteins remained on the beads, as shown by their release via boiling in SDS-PAGE sample buffer (lane B). In subsequent experiments the D-biotin elution step was omi [file ppat.1010362.s008.pdf]

S8 Fig

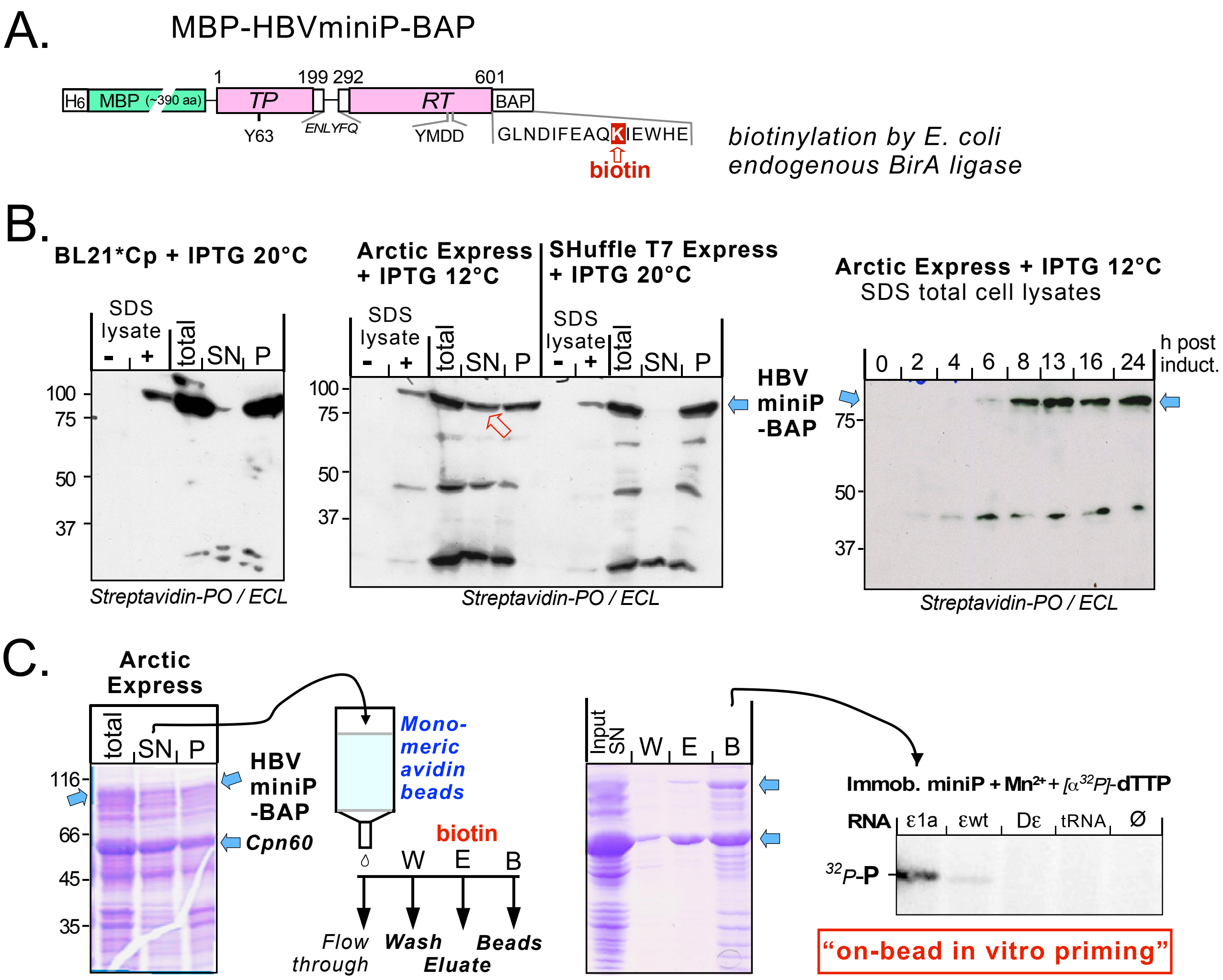

S8 Fig. Native preparation of immobilized recombinant MBP-HBV-miniP-BAP protein. (A) Domain structure of the MBP-HBV-miniP-BAP protein. The protein is identical to MBP-HBV-miniP-H6 (S5 Fig) except that the C terminal His_6 tag is replaced by a Biotin-Acceptor-Peptide (BAP; also known as Avi-tag); its central K residue can be biotinylated by *E. coli* BirA biotin ligase. **(B) Expression testing.** Plasmid pET-MBP-HP1-199_292-601-BAP encoding the MBP-HBV-miniP-BAP ORF under control of the phage T7 RNA polymerase promoter was transformed into BL21*Cp cells, Arctic Express (AE) cells (Agilent), or SHuffle T7 Express cells (NEB); note that AE cells express large amounts of the cold shock chaperonins Cpn60 (*arrow*) and Cpn10. After induction using 0.5 mM IPTG BL21*Cp and SHuffle Express cells were shaken at 20°C and AE cells at 12°C overnight. Small aliquots of cells without vs. with IPTG were directly lysed in SDS-PAGE sample buffer (SDS lysate -/+); the bulk of cells was lysed as described in S5 Fig and aliquots of the crude lysate (total) and the supernatant (SN) and pellet (P) after centrifugation were analzyed by SDS-PAGE and subsequent immunoblotting with peroxidase (PO) conjugated streptavidin, followed by enhanced chemiluminescent (ECL) substrate. Only the SN sample from AE sample contained a reactive ~100 kDa band. **(C) Enrichment of immobilized MBP-HBV-miniP-BAP protein.** Cleared lysate from the induced AE culture was passed through a small column (5 ml volume for lysate from a 300 ml culture) filled with Pierce monomeric avidin agarose (Thermo Scientific). Column preparation, sample loading and washing (lane W) were performed as recommended by the manufacturer. A fraction of the 100 kDa protein could be eluted (lane E), together with excess Cpn60, by 2 mM D-biotin in wash buffer (100 mM Na^+ phosphate, 150 mM NaCl, pH 7.0). More of the 100 kDa protein plus Cpn60 and various less abundant proteins remained on the beads, as shown by their release via boiling in SDS-PAGE sample buffer (lane B). In subsequent experiments the D-biotin elution step was omitted. Instead the washed beads were stored, after adding EDTA-free protease inhibitor cocktail (Roche), in small aliquots at -80° C. For on-bead in vitro priming, about 5 μl beads (~200 ng miniP) were adjusted in a total volume of 10 μl to 25 mM Tris/Cl^- [pH 8.0], 2.5 mM DTT, 5 mM $MgCl_2$, 1 U/μl RNasin (Promega) and typically 1 μM ε RNA. After 1 h at 37°C 5 μl priming mix were added as described in Fig 5 for renatured HBV-miniP protein.
